# Supplementary material for: Identifying immune signatures of sepsis to increase diagnostic accuracy in very preterm babies
Source: Nat Commun. 2024 Jan 9;15:388. doi: 10.1038/s41467-023-44387-5 (PMC10776581; doi:10.1038/s41467-023-44387-5)
Supplement: Supplementary file 1 — Supplementary Information [file 41467_2023_44387_MOESM1_ESM.pdf]

Supplementary Information

Identifying immune signatures of sepsis to increase diagnostic accuracy in very preterm babies

Das et al.

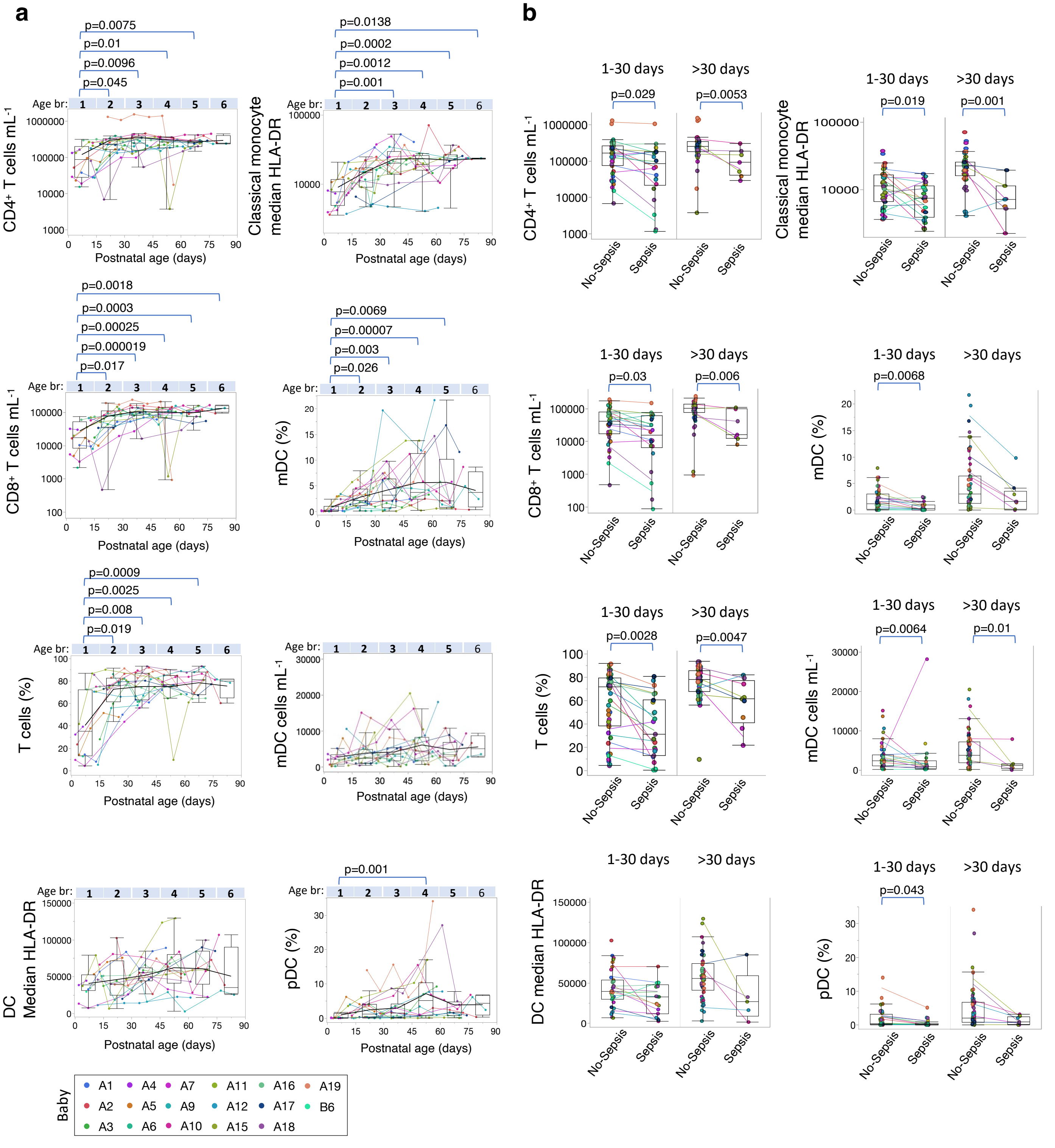

Supplementary Figure 1. **Postnatal age-related changes in immune parameters**. **a**, Each parameter from Fig 1e. is shown over time (No-Sepsis samples only). Lines connect all longitudinal samples from an individual baby. Line colours refer to individual babies. Samples are additionally grouped into six age brackets (Age br. 1= postnatal days 1-15 (n=20 from 14 babies); 2= 16-30 days (n=24 from 15 babies); 3= 31-45 days (n=22 from 13 babies); 4= 46-60 days (n=18 from 11 babies); 5= 61-75 days (n=16 from 9 babies); 6= 76-90 days (n=4 from 3 babies)). Thick black line shows the mean at each age bracket. Box plots show median, first quartile (Q1) and 3rd quartile (Q3) and whiskers extend between Q1-1.5 x IQR (interquartile range) and Q3+1.5 x IQR. *P*-values were generated by a Kruskal-Wallis test with Dunn's post hoc correction. **b**, Comparison of Sepsis versus No-Sepsis samples after stratification by age into two groups (1-30 days: Sepsis n=23 samples from 17 babies; No-Sepsis n=44 samples from 17 babies. >30 days: Sepsis n=9 from 8 babies; No-Sepsis n=60 samples from 14 babies). Duplicate sepsis samples from the same episode were excluded as shown in Fig. 1c. Lines connect the mean of all No-Sepsis samples from an individual baby with the mean of all Sepsis samples from the same baby. Line colours refer to individual babies. *P*-values were generated by a two-tailed Mann-Whitney test. Box plots show median, first quartile (Q1) and 3rd quartile (Q3) and whiskers extend between Q1-1.5 x IQR (interquartile range) and Q3+1.5 x IQR. N may vary slightly between graphs due to experimental dropouts or data filtering (see methods).

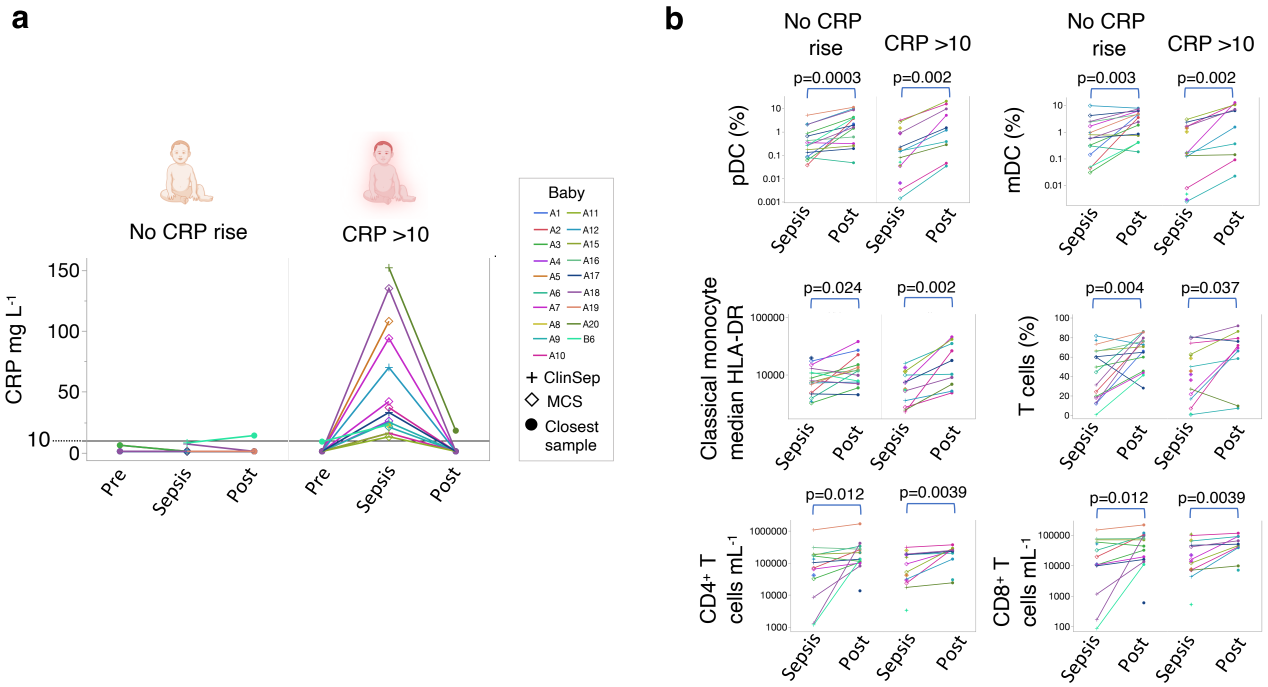

Supplementary Figure 2. Temporal sepsis-induced perturbations. **a**, Sepsis episodes were differentiated into those accompanied by a CRP rise >10mg L^{-1} (n=16 episodes from 13 babies) or those with no CRP rise; ≤10mg L^{-1} (n=16 episodes from 12 babies). Lines connect sepsis samples to those obtained approximately one week before and after sepsis, from the same baby. Figure created with BioRender.com. **b**, Key immune parameters were compared between paired sepsis and post-sepsis timepoints obtained approximately one week apart, now shown within 'No CRP rise' and 'CRP >10' sub-cohorts separately. Statistical analysis was performed using two-sided Wilcoxon matched-pairs signed-rank test. **a-b**, Line colours denote which baby the sepsis episode belongs to; babies may have had more than one episode of sepsis (n=32 episodes of sepsis from 19 babies). N may vary slightly between graphs due to experimental dropouts or data filtering (see methods).

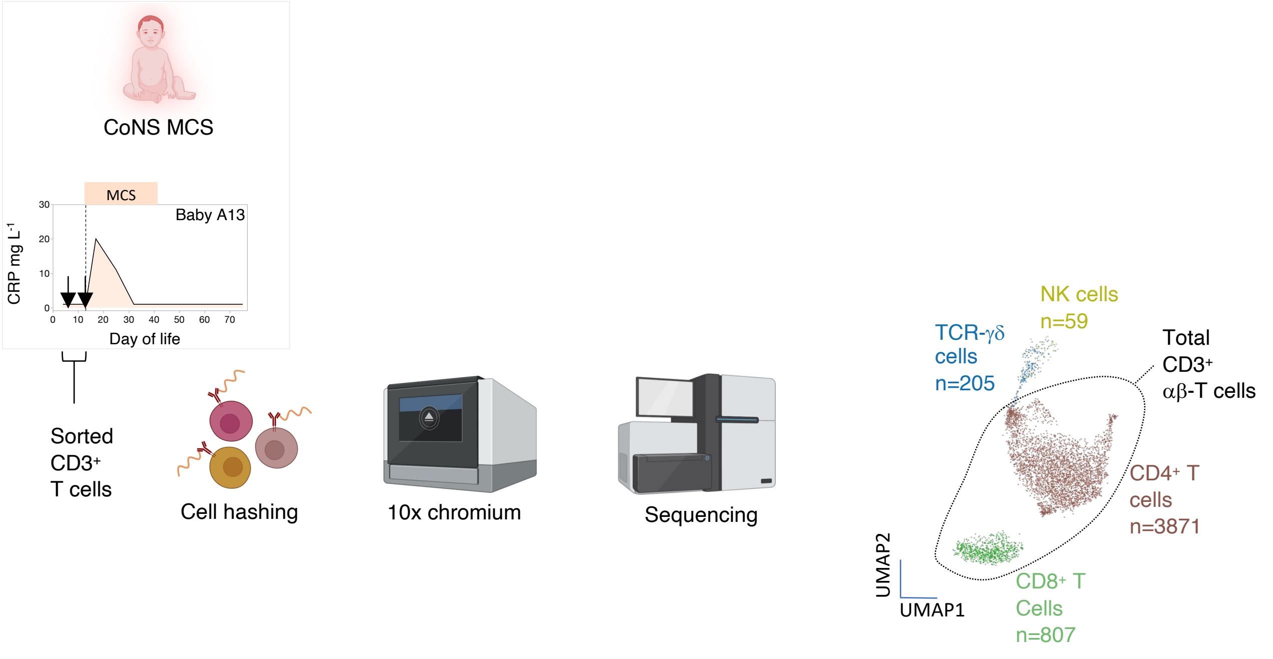

Supplementary Figure 3. scRNA-seq of sorted CD3⁺ T cells during CoNS MCS. Graph shows temporal changes in CRP over time for baby A13 and black arrows depict when blood was drawn in relation to onset of CoNS MCS (dotted line). ScRNA-seq was performed on sorted total CD3⁺ T cells. **b**, UMAP projection of 4,942 cells (2 samples from baby A13). The UMAP and cell clusters are related to Figures 2b-c. Figure created with BioRender.com.

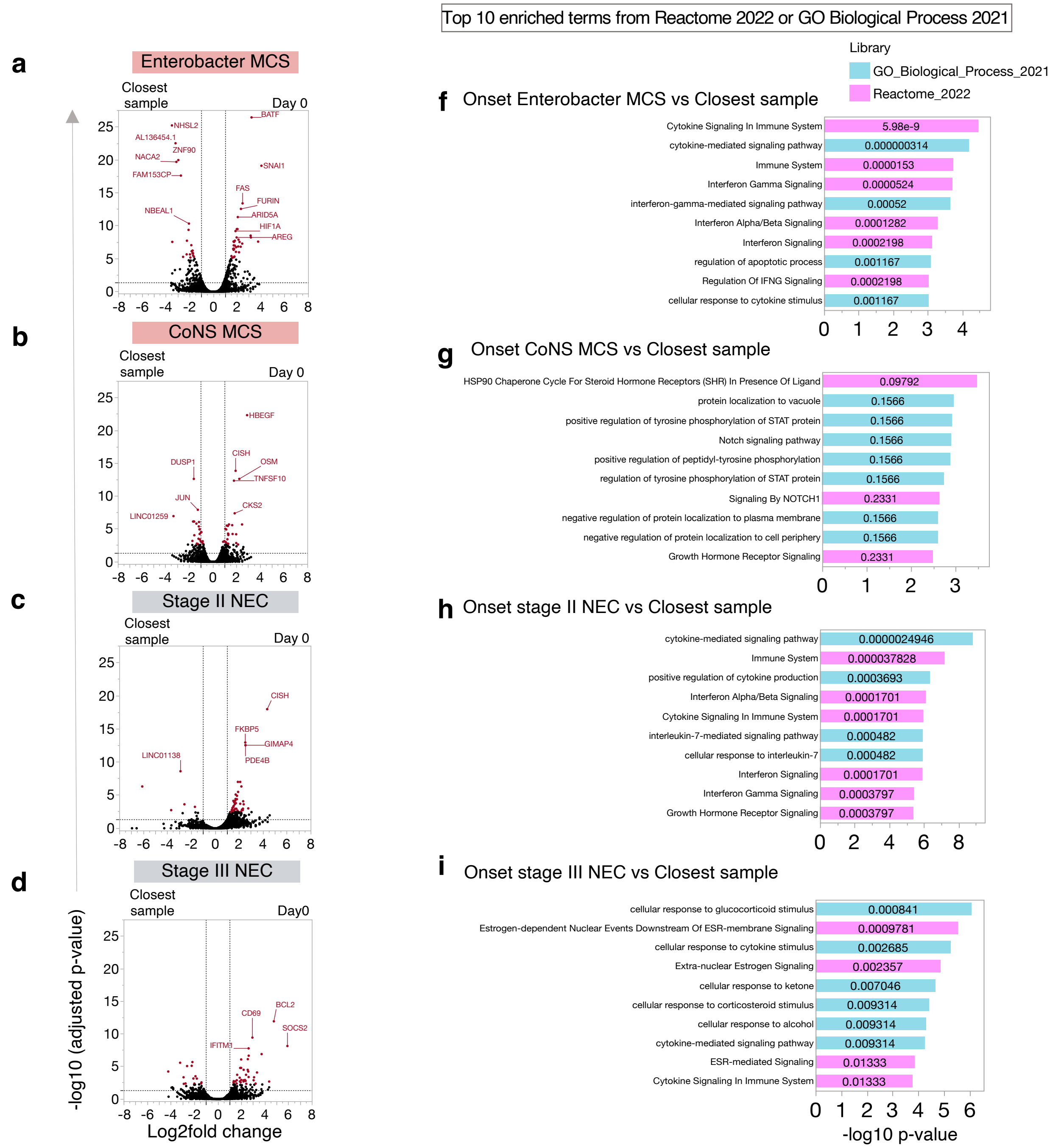

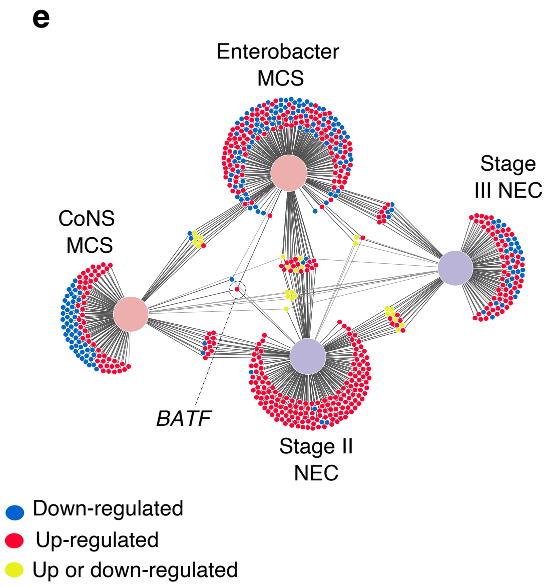

Supplementary Figure 4. CD3⁺ αβ-T cell signatures during MCS and NEC. Volcano plots show differential gene expression (determined with the differential expression algorithm in Loupe Browser (10X genomics, v5.1.0), which is based on the negative binomial exact test used in the sSeq method) between CD3⁺ αβ-T cells obtained at onset (day 0) of **a**, Enterobacter MCS; **b**, CoNS MCS; **c**, Stage II NEC or **d**, Stage III NEC versus the temporally closest blood sample from the same baby (see Fig. 2a and Supplementary Fig. 3 for timing of sampling). Top 50 DEG are shown in maroon; selected DEG are annotated. **e**, Venn diagram (created in DiVenn 1.2) compares all DEGs (Benjamini-Hochberg (BH) adjusted p-values <0.05, **I**log2fold change**I** > 0.95) between the four disease responses in **a-d**. Pathway analysis of all upregulated DEGs (BH adjusted p-value <0.05; log2foldchange >0.95) during **f**, Enterobacter MCS; **g**, CoNS MCS; **h**, Stage II NEC and **i**, stage III NEC. Bar graphs depict the top ten enriched terms from the Reactome 2022 gene set library (pink bars) or GO Biological Processes 2021 library (turquoise bars). P-values (Fisher exact test), adjusted for multiple testing using the Benjamini-Hochberg method, are annotated on each bar. Haemaglobin genes (*HBB, HBA1, HBA2, HBG1, HBG2, HBD, HBE1, HBM, HBQ1, HBZ*) were filtered prior to making all graphs.

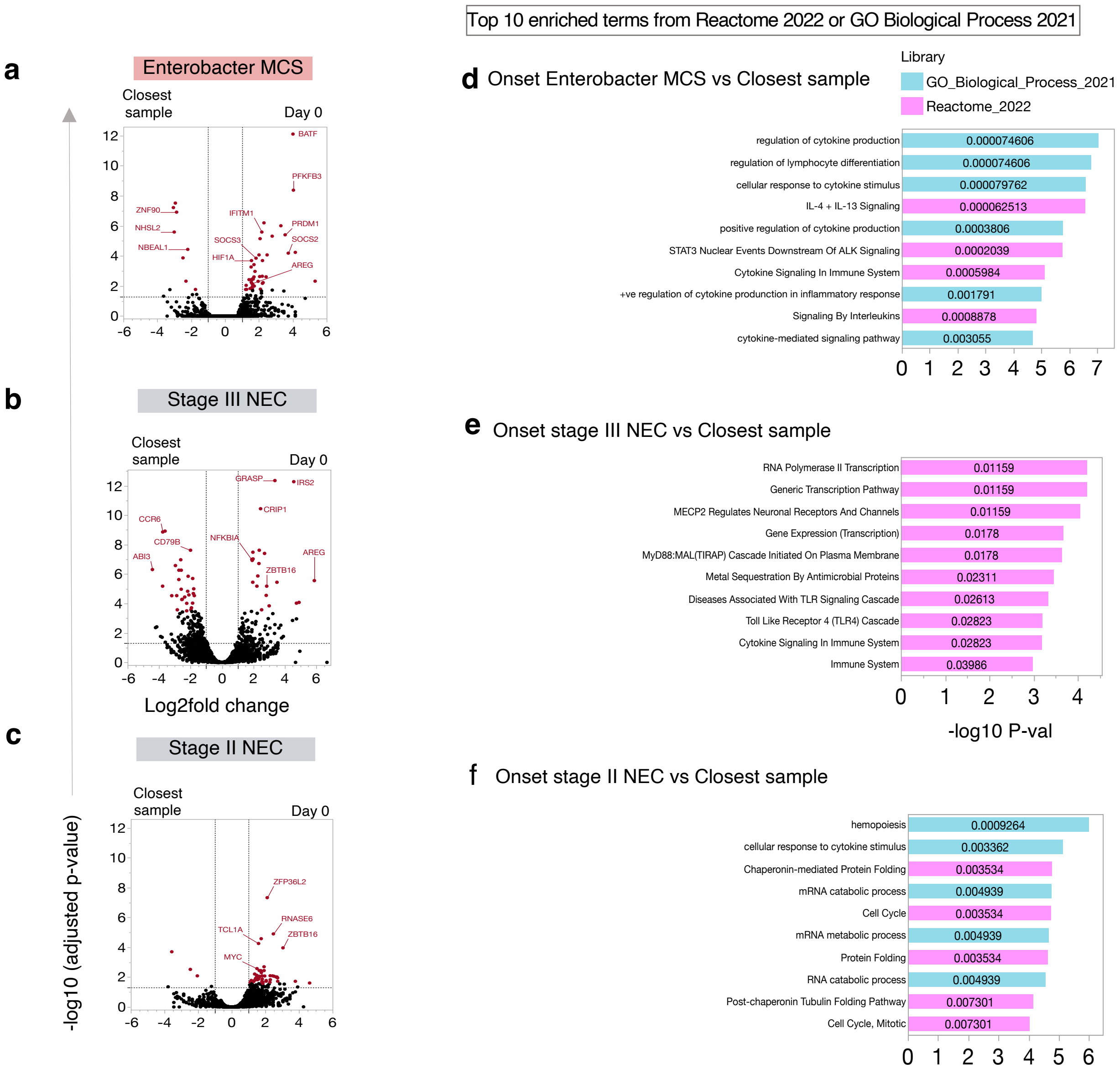

Supplementary Figure 5. B cell signatures during MCS and NEC. Volcano plots show differential gene expression (determined with the differential expression algorithm in Loupe Browser (10X genomics, v5.1.0), which is based on the negative binomial exact test used in the sSeq method) between B cells obtained at onset (day 0) of **a**, Enterobacter MCS; **b**, Stage III NEC or **c**, Stage II NEC versus the temporally closest blood sample from the same baby (see Fig. 2a for timing of sampling). Top 50 DEGs are shown in maroon; selected DEGs are annotated. **d-f**. Pathway analysis of all upregulated DEGs (BH adjusted p-value <0.05; log2foldchange >0.95) during **d**, Enterobacter MCS; **e**, Stage III NEC and **f**, stage II NEC. Bar graphs depict the top ten enriched terms from the Reactome 2022 gene set library (pink bars) or GO Biological Processes 2021 library (turquoise bars). P-values (Fisher exact test), adjusted for multiple testing using the Benjamini-Hochberg method, are annotated on each bar. Haemaglobin genes (*HBB, HBA1, HBA2, HBG1, HBG2, HBD, HBE1, HBM, HBQ1, HBZ*) were filtered prior to making all graphs.

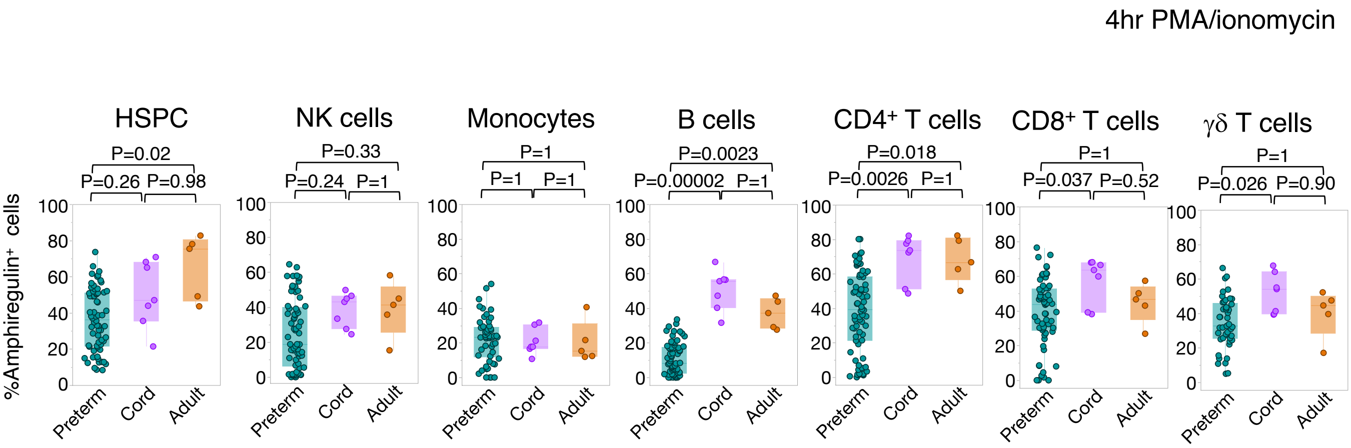

Supplementary Figure 6. Frequency of amphiregulin+ haematopoeitic stem and progenitor cells (HSPC), NK cells, monocytes, B cells, CD4+ T cells, CD8+ T cells and γδ T cells after mitogen activation of PBMC comparing samples obtained from preterm infants (n=72 samples from 11 patients), umbilical cord blood at term (n=7) and adult peripheral blood (n=5). Box plots; central line denotes the median, upper and lower lines of box represent the 75th and 25th percentiles respectively and whiskers show the range (minimum to maximum value). *P*-values were generated by a Kruskal-Wallis test with Dunn's post hoc correction. PMA = Phorbol 12-myristate 13-acetate.

Dependent variable	% T cells				
Fixed effects	Estimate	Lower 95% CI	Upper 95% CI	Standard Error	P value
Intercept	31.99419	-108.0443	172.03268	62.85231	0.62176554
Sepsis (Yes)	-8.53968	-12.53865	-4.540708	2.018843	0.000047
Postnatal age	2.906479	1.8011131	4.0118443	0.558098	0.0000008
Sex (Female)	-5.811171	-17.68533	6.0629861	5.428653	0.30619056
Gestational age at birth	0.034728	-0.761188	0.8306445	0.356436	0.924339052
Birth weight Z-score	-1.602624	-8.856251	5.6510026	3.256415	0.633219814
Respiratory Support	8.919838	-2.356141	20.195817	5.183401	0.110574675
Antenatal Steroids	-5.437445	-15.21687	4.3419789	4.486615	0.248946646

Dependent variable	CD8+ T cells/ml blood				
Fixed effects	Estimate	Lower 95% CI	Upper 95% CI	Standard Error	P value
Intercept	-35573.66	-371879.4	300732.1	154113.9	0.821391273
Sepsis (Yes)	-11906.22	-19999.32	-3813.114	4084.327	0.004
Postnatal age	5735.897	3553.5024	7918.2909	1101.445	0.00000087
Sex (Female)	-8136.104	-36227.4	19955.189	13065.35	0.543734912
Gestational age at birth	392.1201	-1520.702	2304.9426	875.4187	0.662390257
Birth weight Z-score	5059.605	-12382.6	22501.806	7990.74	0.53868651
Respiratory Support	10623.21	-16088.98	37335.394	12437.27	0.407612135
Antenatal Steroids	-5210.159	-28276.87	17856.548	10757.23	0.635615875

Dependent variable	% mDC				
Fixed effects	Estimate	Lower 95% CI	Upper 95% CI	Standard Error	P value
Intercept	-16.64747	-38.45475	5.1598231	9.99927	0.122030189
Sepsis (Yes)	-1.114541	-1.822114	-0.406968	0.357274	0.0023
Postnatal age	0.437371	0.2419628	0.6327781	0.098678	0.00002
Sex (Female)	-0.548563	-2.418445	1.3213198	0.869971	0.538717635
Gestational age at birth	0.09671	-0.027105	0.2205237	0.056653	0.114232787
Birth weight Z-score	0.239291	-0.890068	1.3686495	0.5182	0.652522962
Respiratory Support	-0.202745	-1.98236	1.5768713	0.833343	0.81114731
Antenatal Steroids	-0.757701	-2.301757	0.7863557	0.720312	0.310557293

Dependent variable	%pDC				
Fixed effects	Estimate	Lower 95% CI	Upper 95% CI	Standard Error	P value
Intercept	-28.60106	-57.90302	0.7008962	13.43938	0.054871969
Sepsis (Yes)	-0.966306	-1.7575	-0.175111	0.399461	0.017
Postnatal age	0.381818	0.163079	0.6005572	0.110447	0.00076
Sex (Female)	-0.324194	-2.798619	2.1502306	1.151996	0.782569222
Gestational age at birth	0.164344	-0.002202	0.3308905	0.076265	0.05263378
Birth weight Z-score	0.264264	-1.254275	1.7828031	0.696446	0.711029122
Respiratory Support	0.172495	-2.18045	2.5254402	1.099002	0.877478676
Antenatal Steroids	-0.349756	-2.385737	1.6862251	0.950538	0.718332307

Dependent variable	Classical monocytes median HLA-DR				
Fixed effects	Estimate	Lower 95% CI	Upper 95% CI	Standard Error	P value
Intercept	-21307.88	-71576.67	28960.914	23190.32	0.375463359
Sepsis (Yes)	-2384.799	-4431.91	-337.6883	1032.498	0.023
Postnatal age	1634.868	1068.7536	2200.983	285.5665	0.00000009
Sex (Female)	2557.196	-1801.47	6915.8616	2034.215	0.229076789
Gestational age at birth	163.4594	-121.5722	448.49098	131.1628	0.235909928
Birth weight Z-score	1441.972	-1150.068	4034.0108	1193.7	0.24962455
Respiratory Support	-1862.083	-6003.42	2279.2539	1939.217	0.352497779
Antenatal Steroids	-2.354605	-3623.116	3618.4073	1695.975	0.998910892

Dependent variable	Amphiregulin pg/ml				
Fixed effects	Estimate	Lower 95% CI	Upper 95% CI	Standard Error	P value
Intercept	257.9517	-328.0711	843.97457	267.0034	0.3543
Sepsis (Yes)	22.60128	0.6423063	44.56025	11.107	0.04
Postnatal age	-15.33985	-23.33515	-7.344551	4.047324	0.0002
Sex (Female)	-20.55683	-52.16165	11.047989	14.44363	0.1811
Gestational age at birth	-0.591877	-3.889708	2.7059534	1.495656	0.7
Birth weight Z-score	-7.494781	-25.28903	10.299468	8.010502	0.371

Supplementary Figure 7. **Multivariate analysis to identify potential confounders.** Data were analysed by a linear mixed-effects model fit via REML (Restricted Maximum Likelihood). Baby ID was set as a random effect to account for repeated measures. Dependent variables: % T cells, $CD8^+$ T cell numbers, %mDC, %pDC, classical monocyte median HLA-DR and plasma amphiregulin. Fixed effects: Sepsis (Yes); postnatal age; female sex; gestational age; birthweight Z-score. Respiratory support and antenatal steroids were included for all parameters except amphiregulin due to missing data. Flow cytometry cohort: Sepsis n=32, No-Sepsis n=104 (see Fig. 1c). Amphiregulin cohort: Sepsis n=47, No-Sepsis n=116 (see Fig. 6b). CI= confidence interval.

a

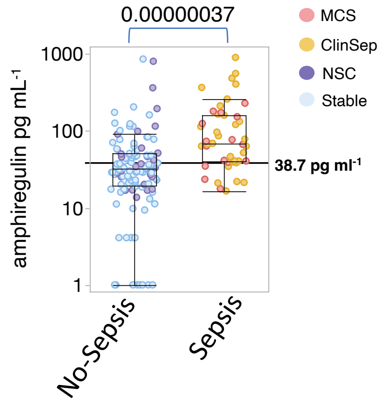

b

Samples obtained within 48 hours of blood culture for suspected sepsis

	amphiregulin	CRP
Prevalence	0.54	0.54
Sensitivity	0.88	0.55
Specificity	0.54	1
PPV	0.69	1
NPV	**0.79**	**0.65**

c

CRP			amphiregulin			CRP and amphiregulin		
Prior probability of infection	PPV	NPV	Prior probability of infection	PPV	NPV	Prior probability of infection	PPV	NPV
0	-	1	0	0	1	0	0	1
0.01	1	1	0.01	0.02	1	0.01	0.02	1
0.05	1	0.98	0.05	0.09	0.99	0.05	0.1	1
0.1	1	0.95	0.1	0.18	0.98	0.1	0.19	0.99
0.15	1	0.93	0.15	0.25	0.96	0.15	0.27	0.99
0.2	1	0.9	0.2	0.32	0.95	0.2	0.35	0.99
0.25	1	0.87	0.25	0.39	0.93	0.25	0.41	0.98
0.3	1	0.84	0.3	0.45	0.91	0.3	0.47	0.98
0.4	1	0.77	0.4	0.56	0.87	0.4	0.58	0.96
0.5	1	0.69	0.5	0.66	0.82	0.5	0.68	0.95
0.6	1	0.6	0.6	0.74	0.75	0.6	0.76	0.92
0.7	1	0.49	0.7	0.82	0.66	0.7	0.83	0.89
0.75	1	0.43	0.75	0.85	0.6	0.75	0.86	0.86
0.8	1	0.36	0.8	0.88	0.53	0.8	0.89	0.82
0.85	1	0.28	0.85	0.92	0.44	0.85	0.92	0.76
0.9	1	0.2	0.9	0.95	0.33	0.9	0.95	0.67
0.95	1	0.1	0.95	0.97	0.19	0.95	0.98	0.49
0.99	1	0.02	0.99	0.99	0.04	0.99	1	0.15
1	1	0	1	1	0	1	1	0

Supplementary Figure 8. Evaluating a test for sepsis based on amphiregulin and CRP. a, Amphiregulin in No-Sepsis (116 samples from 23 babies) versus Sepsis (46 samples from 23 babies) samples. Horizontal line represents the threshold of the maximal Youden index (38.7 pg mL^{-1}), identified from analysis of all samples. Box plot shows median, first quartile (Q1) and 3rd quartile (Q3) and whiskers extend between Q1-1.5 x IQR (interquartile range) and Q3+1.5 x IQR. P-value was generated by a two-tailed Mann–Whitney test. **b**, Sensitivity, specificity, positive predictive value (PPV) and negative predictive value (NPV) of amphiregulin alone (cutoff >38.7 pg mL^{-1}) or CRP alone (>10 mg L^{-1}) to diagnose/rule-out sepsis. Analysis restricted to samples obtained within 48 hours of a suspected sepsis episode (NSC n=28 from 17 babies; Sepsis (ClinSep+MCS) n=33 from 20 babies). Duplicate samples from the same sepsis episode were excluded as shown in Fig. 6b. Prevalence in this setting = (ClinSep+MCS)/(ClinSep+MCS+NSC); the number of suspected sepsis samples that were subsequently classified as MCS or ClinSep. **c**, Table (relating to Fig. 6e and Supplementary Fig. 8b) summarizes positive and negative predictive values (PPV and NPV) for tests based on CRP alone, amphiregulin alone, or both together, across a range of prior probabilities of infection. Table generated using Epitools Calculator available at: http://epitools.ausvet.com.au.

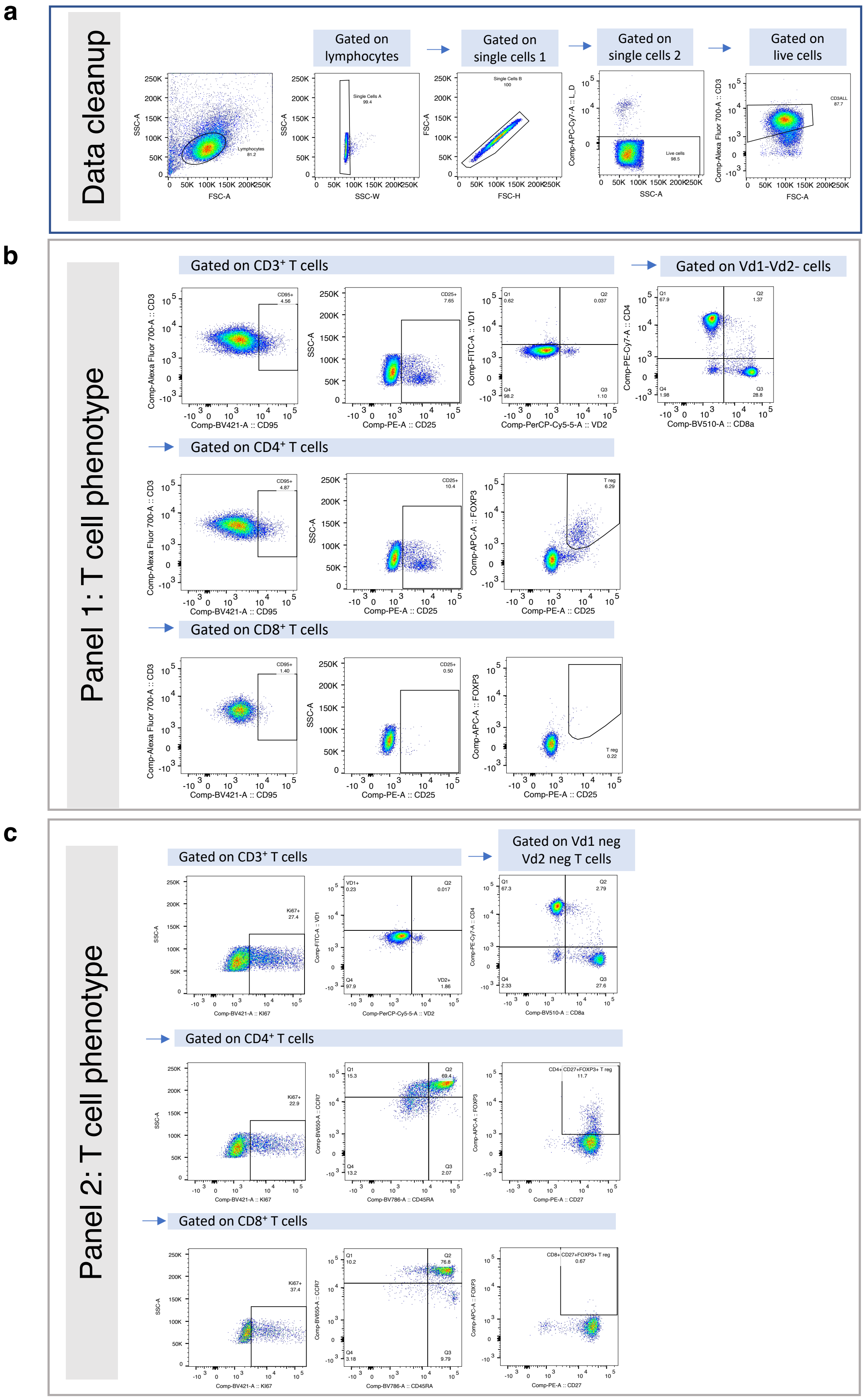

Supplementary Figure 9: Flow cytometry gating strategies for panels 1 and 2. a, Data clean-up and a shared initial gating workflow is shown. **b**, Panel 1: Gating strategy used to examine frequencies of Vd1, Vd2, $CD4^+$ and $CD8^+$ T cells, $CD4^+$ T regs, $CD8^+$ $FOXP3^+CD25^+$ T cells and frequencies of T cells expressing CD25 or CD95/FAS. **c**, Panel 2: Gating strategy to examine frequencies of proliferating ($Ki67^+$) cells, naïve and memory cells as well as $CD27^+FOXP3^+$ cells within $CD4^+$ and $CD8^+$ T cells.

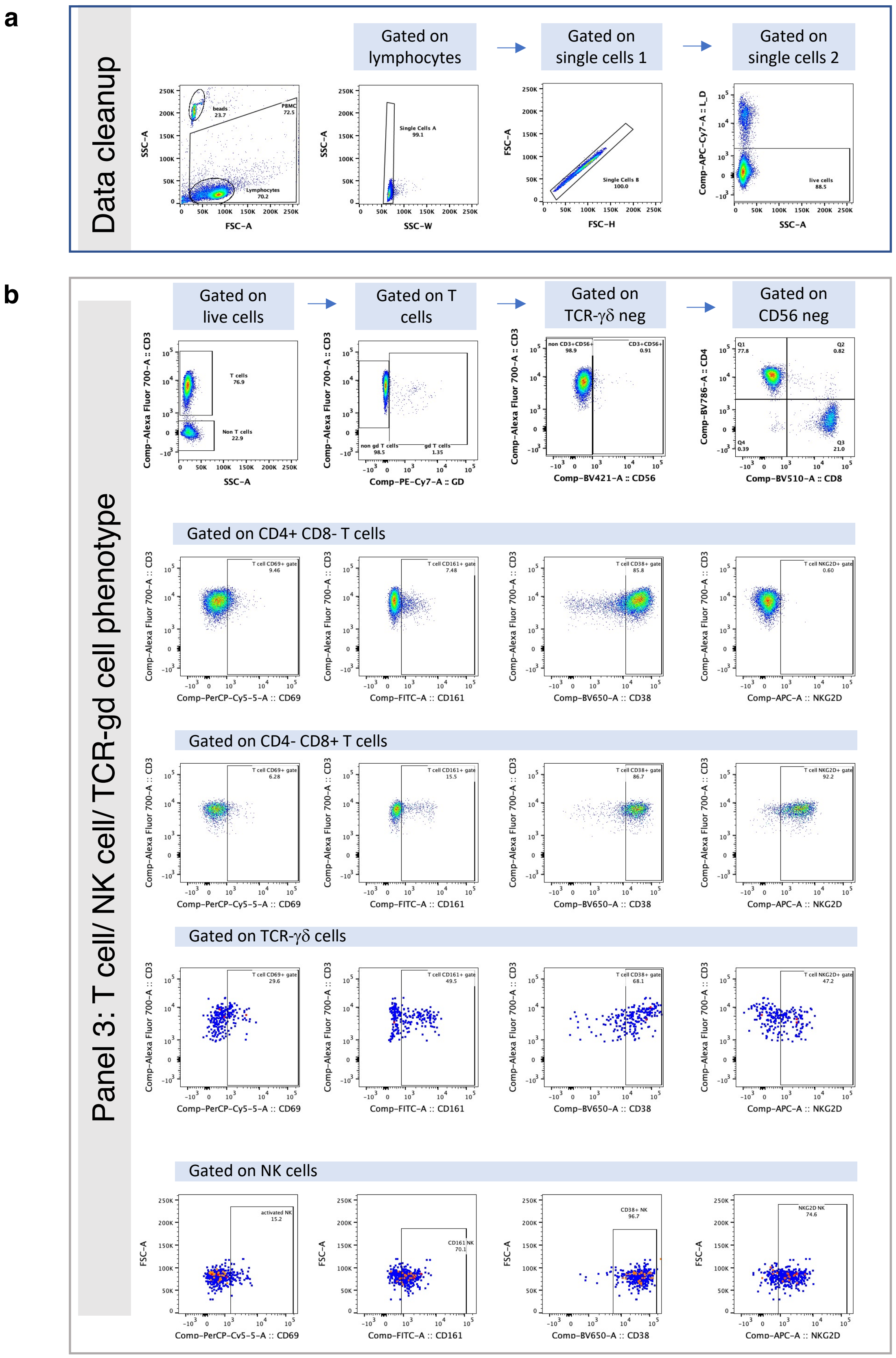

Supplementary Figure 10: Flow cytometry gating strategy for panel 3. a, Data clean-up and a shared initial gating workflow is shown. **b**, Gating strategy to examine expression of CD161, CD38, NKG2D and CD69 on T cells, NK and TCR-γδ cells.

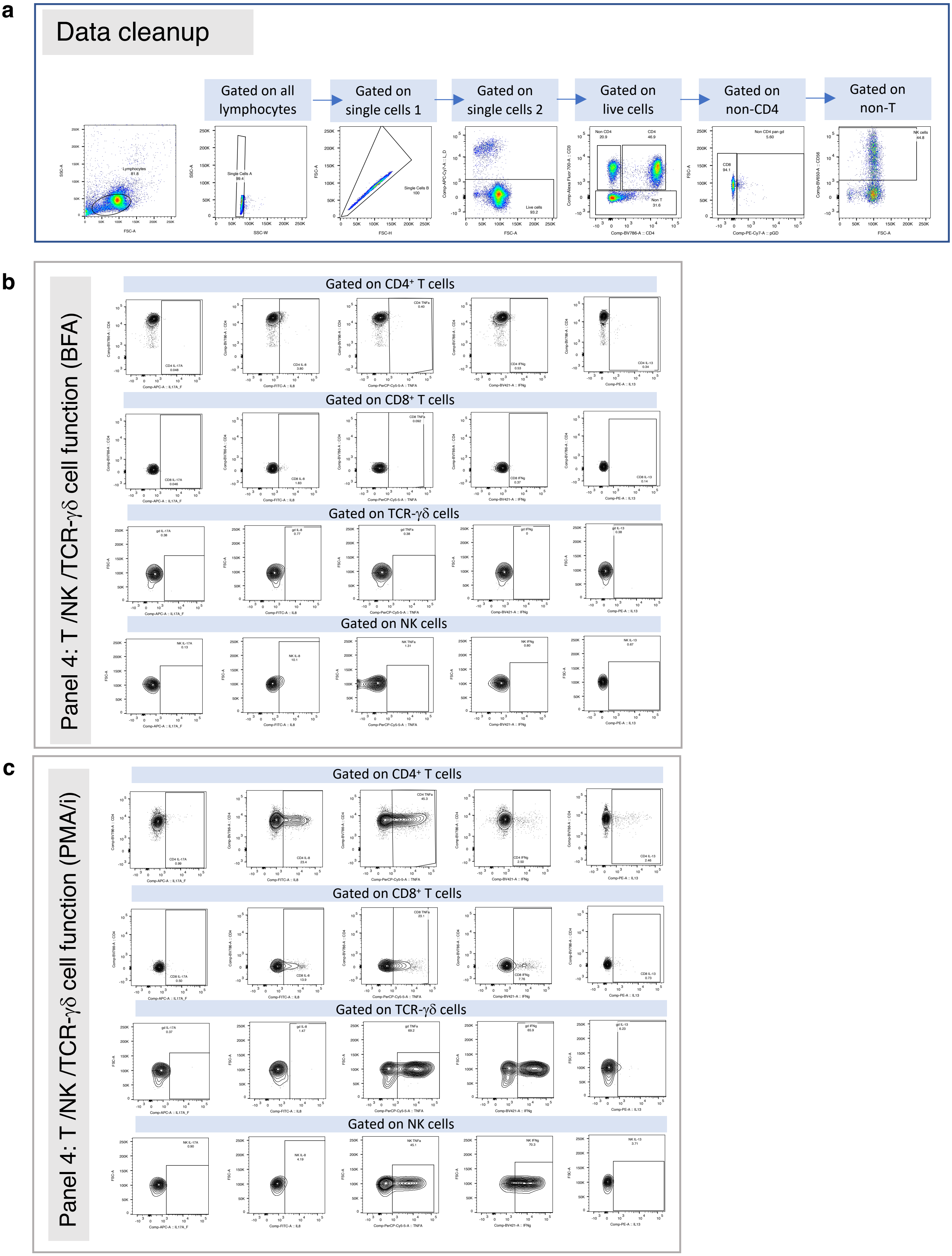

Supplementary Figure 11: Flow cytometry gating strategy for panel 4. a, Data clean-up and a shared initial gating workflow is shown. Gating strategy to examine production of CXCL8, TNFα, IL-17A/F, IFNγ and IL-13 within T cells, NK and TCR-γδ cells by intracellular cytokine staining. Cells were incubated with **b**, Brefeldin A (BFA) only to demonstrate spontaneous cytokine production, or **c**, BFA in addition to PMA and ionomycin to determine cytokine production after stimulation.

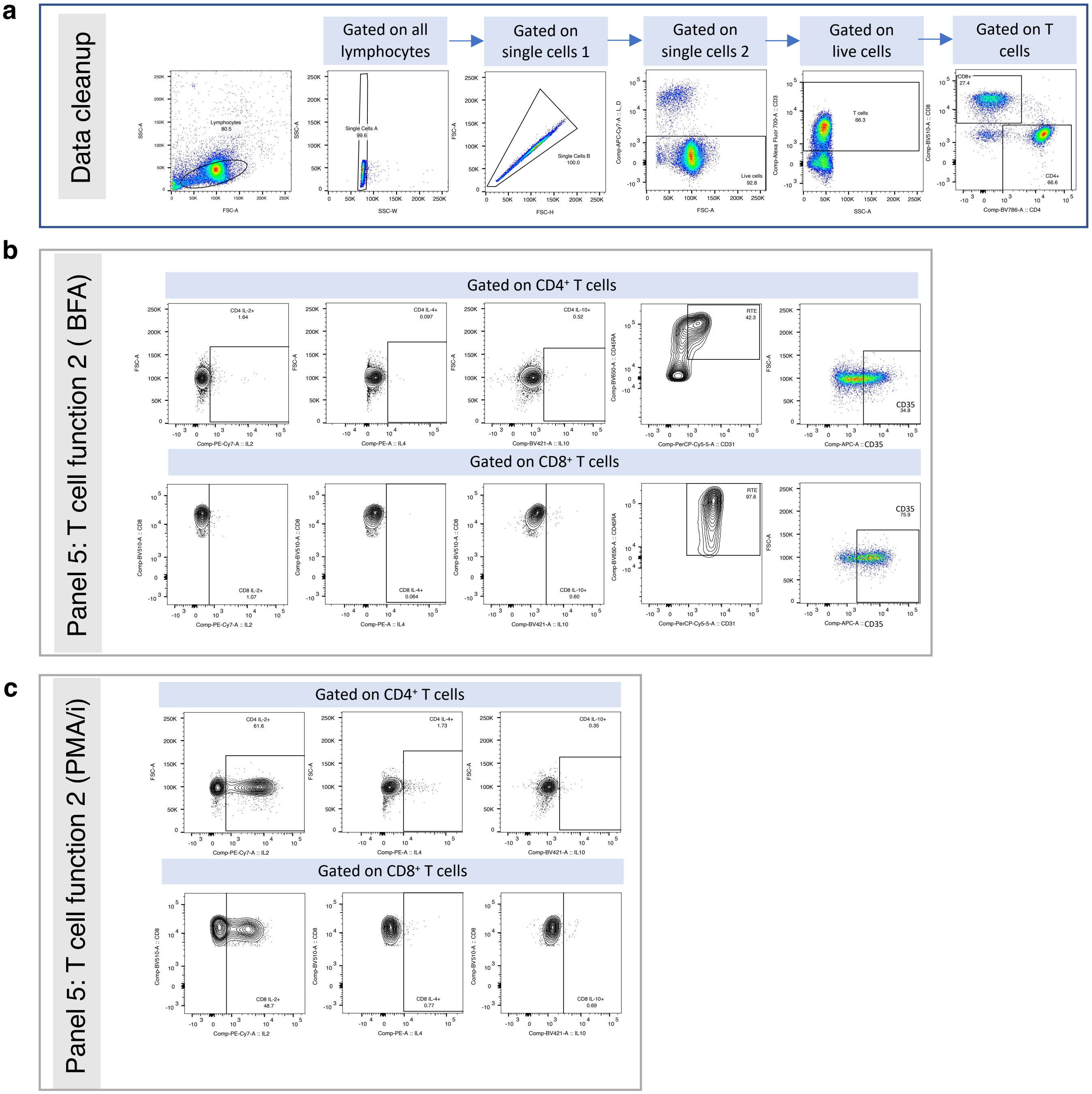

Supplementary Figure 12: Flow cytometry gating strategy for panel 5. a, Data clean-up and an initial gating workflow is shown. IL2, IL4 and IL-10 production by intracellular cytokine staining in $CD4^+$ and $CD8^+$ T cells. Cells were incubated with **b**, Brefeldin A (BFA) only to demonstrate spontaneous cytokine production, or **c**, BFA in addition to PMA and ionomycin to determine cytokine production after stimulation. Additionally, for all BFA treated samples, frequencies of recent thymic emigrant (RTE) and $CD35^+$ $CD4^+$ and $CD8^+$ T cells were assessed.

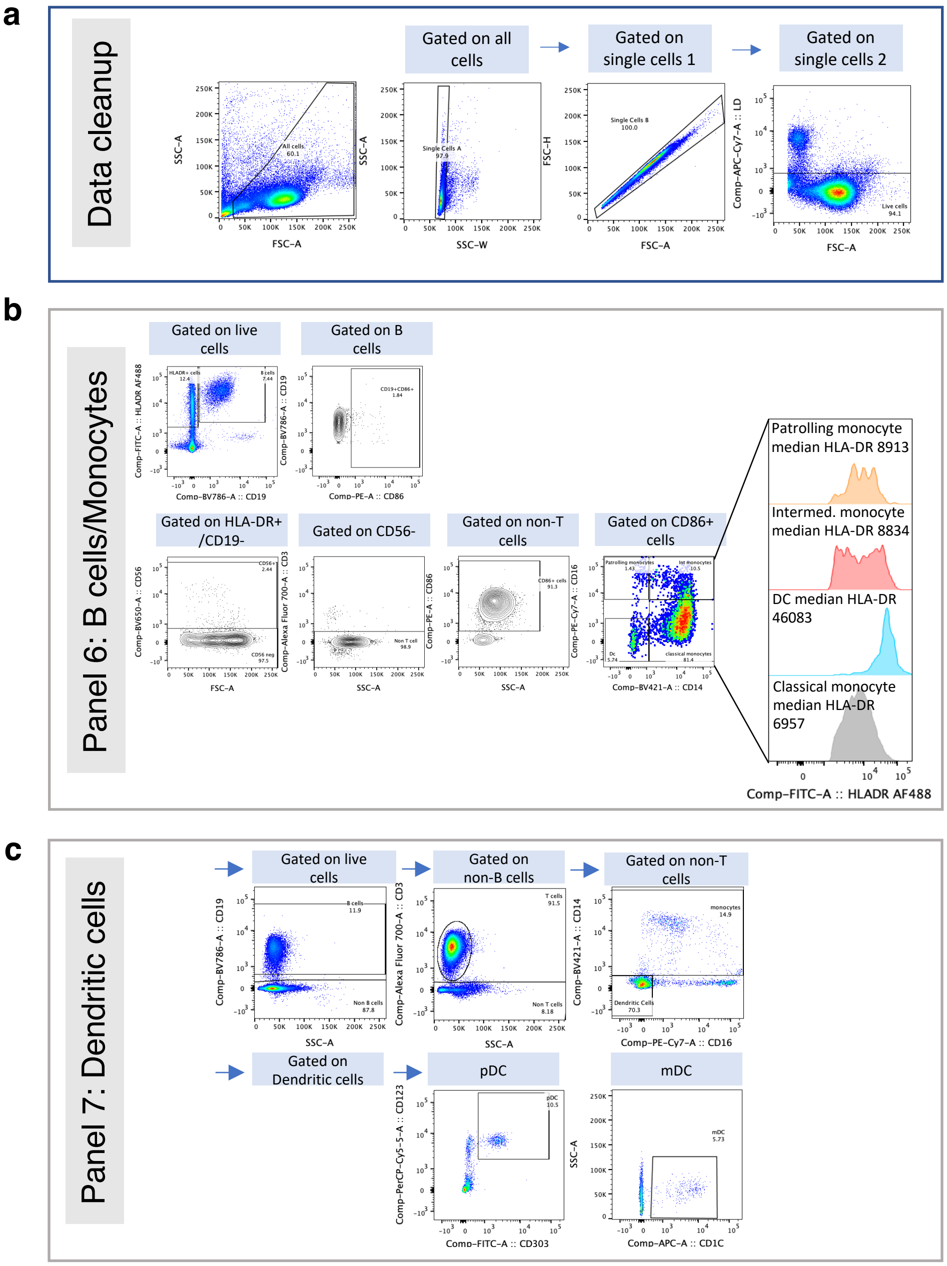

Supplementary Figure 13: Flow cytometry gating strategy for panels 6 and 7. a, Data clean-up and a shared initial gating workflow is shown. Gating strategies to examine **b**, frequencies of B cells and CD86^{+} B cells, monocyte subsets and HLA-DR expression within monocyte subsets or dendritic cells. **c**, Gated strategy to examine pDC and mDC.

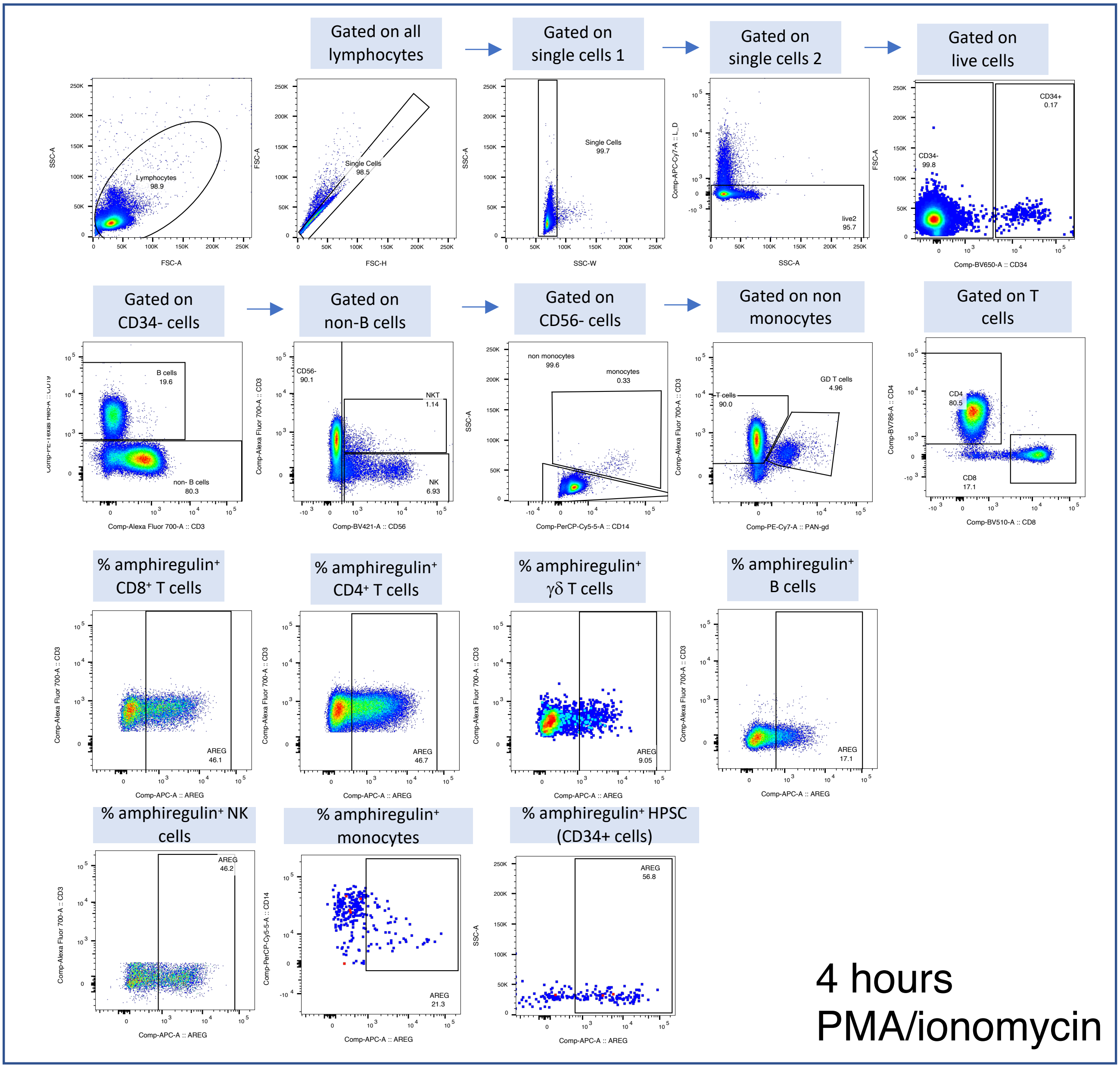

Supplementary Figure 14: Flow cytometry gating strategy for panel 8. Assessment of amphiregulin production within PBMC subsets after activation with PMA and ionomycin in the presence of Brefeldin A (4 hours).

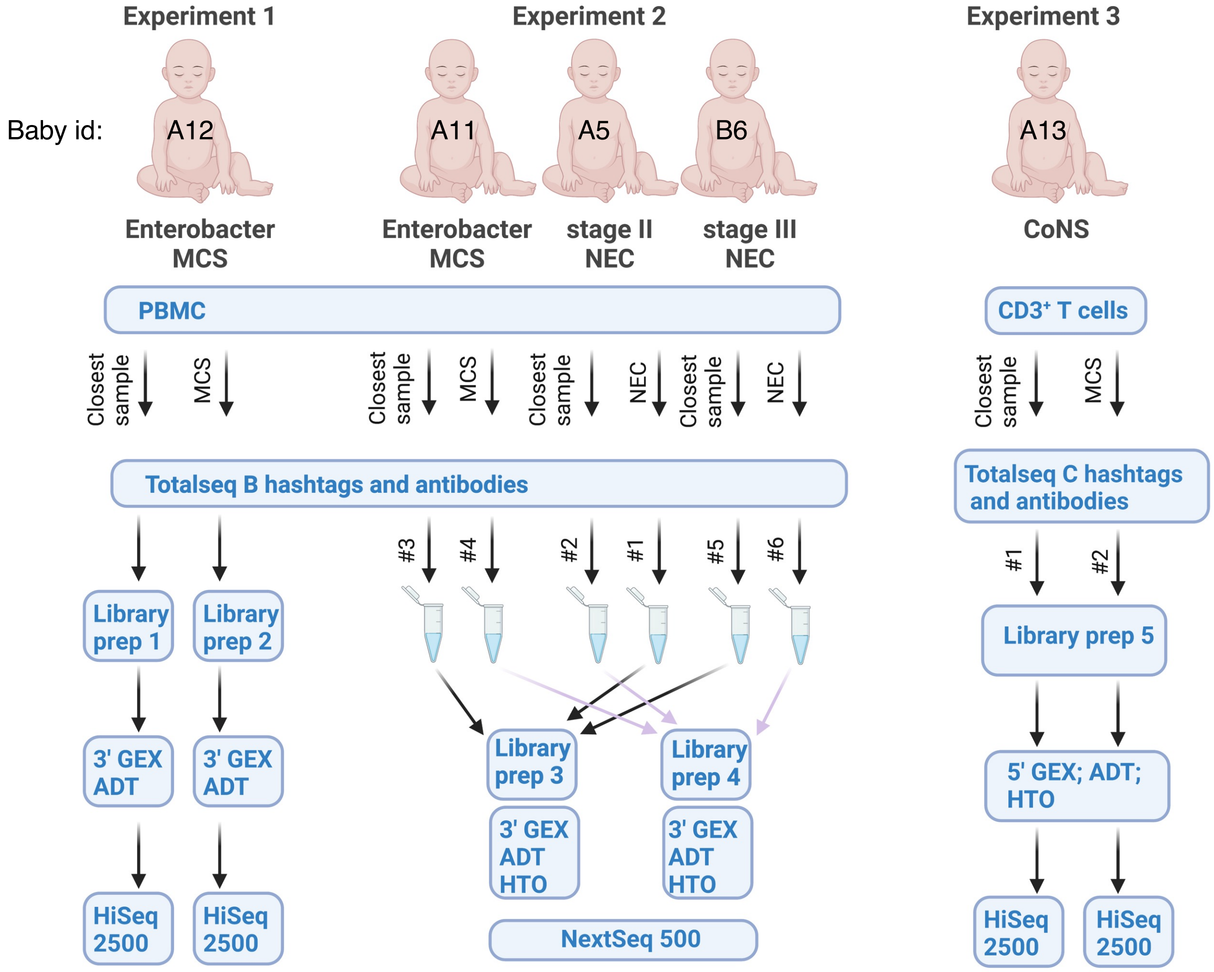

ADT/HTO	Barcode sequence	Baby	Clinical event
TotalSeq™-B0253 anti-human Hashtag 3	TTCCGCCTCTCTTTG	A11	Closest sample
TotalSeq™-B0254 anti-human Hashtag 4	AGTAAGTTCAGCGTA	A11	Enterobacter MCS
TotalSeq™-B0252 anti-human Hashtag 2	TGATGGCCTATTGGG	A5	stage II NEC
TotalSeq™-B0251 anti-human Hashtag 1	GTCAACTCTTTAGCG	A5	Closest sample
TotalSeq™-B0255 anti-human Hashtag 5	AAGTATCGTTTCGCA	B6	stage III NEC
TotalSeq™-B0256 anti-human Hashtag 6	GGTTGCCAGATGTCA	B6	Closest sample
TotalSeq™-C0251 anti-human Hashtag 1	GTCAACTCTTTAGCG	A13	Closest sample
TotalSeq™-C0252 anti-human Hashtag 2	TGATGGCCTATTGGG	A13	CoNS MCS
TotalSeq™-B0139 anti-human TCR γ/δ	CTTCCGATTCATTCA	A5, A11, B6, A12	all
TotalSeq™-B0072 anti-human CD4	TGTTCCCGCTCAACT	A5, A11, B6, A12	all
TotalSeq™-B0080 anti-human CD8a	GCTGCGCTTTCCATT	A5, A11, B6, A12	all
TotalSeq™-C0072 anti-human CD4	TGTTCCCGCTCAACT	A13	all
TotalSeq™-C0080 anti-human CD8a	GCTGCGCTTTCCATT	A13	all
TotalSeq™-C0154 anti-human CD27	GCACTCCTGCATGTA	A13	all

Supplementary Figure 15. Diagram of the scRNA-seq workflow. GEX = gene expression library. ADT = Antibody Derived Tag library. HTO = Hashtag Oligonucleotide. HiSeq 2500= Illumina HiSeq 2500 sequencing system. Nextseq 500 = Illumina Nextseq 500 platform. A table of all Totalseq hashtags and antibodies is shown below. Figure created with BioRender.com.

Supplementary Table 1. Clinical characteristics of samples in the flow cytometry cohort. Related to Figure 1c.

Baby	day of life	sample category	Blood culture	Days from blood culture to research blood draw	CRP on day of research blood draw (mg/L)	Excluded from Fig 1d	Antibiotic 1	Antibiotic 2	Antibiotic 3	Antifungal
A1	7	No Sepsis Confirmed		-	1		Gentamicin	Vancomycin		
A1	13	Stable		-	1		Vancomycin			
A1	16	Microbiology Confirmed Sepsis	*CoNS*	0	1		Ceftazidime	Vancomycin		
A1	20	Microbiology Confirmed Sepsis	*CoNS*	4	1	Excluded	Ceftazidime	Vancomycin		
A1	22	Microbiology Confirmed Sepsis	*CoNS*	6	1	Excluded	Ceftazidime	Vancomycin		
A1	27	Stable		-	1					
A1	34	Stable		-	1					
A1	41	Stable		-	1					
A1	48	Stable		-	1					
A10	2	Microbiology Confirmed Sepsis	*E coli*	1	37		Gentamicin	Benzylpenicillin		
A10	6	Microbiology Confirmed Sepsis	*E coli*	5	1	Excluded	Gentamicin	Ceftazidime		
A10	13	Stable		-	1		Vancomycin	Ceftazidime		
A10	20	No Sepsis Confirmed		-	1		Flucloxacillin	Gentamicin		
A10	35	Stable		-	1					
A10	41	Clinical Sepsis		-	16		Vancomycin	Ceftazidime		
A10	48	Stable		-	1					
A10	55	Stable		-	1					
A10	62	Stable		-	1					
A10	69	No Sepsis Confirmed		-	1		Flucloxacillin	Gentamicin		
A10	76	Stable		-	1					
A11	4	Stable		-	1					
A11	11	Stable		-	1					
A11	18	Stable		-	1					
A11	32	Microbiology Confirmed Sepsis	*E cloacae*	3	13		Meropenem			
A11	41	Microbiology Confirmed Sepsis	*E cloacae*	12	1	Excluded	Meropenem			
A11	46	Stable		-	1		Meropenem			
A11	53	Stable		-	1					
A12	5	Clinical Sepsis		-	1		Gentamicin	Benzylpenicillin		
A12	12	Stable		-	1					
A12	19	Clinical Sepsis		-	70		Ceftazidime	Vancomycin		
A12	26	Stable		-	1					
A12	34	Stable		-	1					
A12	40	Microbiology Confirmed Sepsis	*E cloacae*	0	1		Gentamicin			
A12	47	Microbiology Confirmed Sepsis	*E cloacae*	7	1	Excluded	Meropenem			
A12	54	Stable		-	1		Meropenem			
A12	61	Stable		-	1		Meropenem			
A15	6	Clinical Sepsis		-	1					
A15	13	Stable		-	1					
A15	20	No Sepsis Confirmed			1					
A15	27	Stable		-	1					
A15	29	No Sepsis Confirmed		-	1					
A15	34	Stable		-	1					
A15	41	No Sepsis Confirmed		-	1					
A15	48	Stable		-	1					
A15	54	No Sepsis Confirmed		-	1					
A15	62	Stable		-	1					
A15	70	Stable		-	1					
A15	76	Clinical Sepsis		-	16					
A16	8	Stable		-	1					
A16	15	No Sepsis Confirmed		-	1					
A16	21	Clinical Sepsis		-	1					
A16	29	Stable		-	1					
A16	37	Stable		-	1					
A16	44	Stable		-	1					
A16	50	Stable		-	1					
A16	64	Stable		-	1					
A16	71	Stable		-	1					

Baby	day of life	sample category	Blood culture	Days from blood culture to research blood draw	CRP on day of research blood draw (mg/L)	Excluded from Fig 1d	Antibiotic 1	Antibiotic 2	Antibiotic 3	Antifungal
A17	7	Clinical Sepsis		-	1					
A17	12	Clinical Sepsis		-	1	Excluded				
A17	17	No Sepsis Confirmed		-	1					
A17	25	Stable		-	1					
A17	29	Clinical Sepsis		-	33					
A17	32	Clinical Sepsis		-	1	Excluded				
A17	39	Stable		-	1					
A17	46	Microbiology Confirmed Sepsis	*E faecalis*	5	1					
A17	48	Microbiology Confirmed Sepsis	*E faecalis*	7	1	Excluded				
A17	67	Stable		-	1					
A17	74	Stable		-	1					
A18	5	Clinical Sepsis		-	7		Benzylpenicillin	Gentamicin		
A18	12	Stable		-	1					
A18	19	No Sepsis Confirmed		-	1					
A18	26	Clinical Sepsis		-	1		Flucloxacillin	Gentamicin		
A18	34	Stable			1					
A18	40	No Sepsis Confirmed		-	1					
A18	47	Microbiology Confirmed Sepsis	*CoNS*	1	135					
A18	54	Microbiology Confirmed Sepsis	*CoNS*	8	1	Excluded				
A18	61	Stable		-	1					
A18	69	Stable		-	1					
A19	8	Clinical Sepsis		-	1		Vancomycin	Ceftazidime		
A19	14	Clinical Sepsis		-	1	Excluded	Vancomycin	Ceftazidime		
A19	21	Stable		-	1					
A19	28	Stable		-	1					
A19	35	Stable		-	1					
A19	43	Stable		-	1					
A19	50	Stable		-	1					
A19	56	Stable		-	1					
A2	3	No Sepsis Confirmed		-	6		Gentamicin	Benzylpenicillin		
A2	7	Microbiology Confirmed Sepsis	*CoNS*	1	1		Ceftazidime	Vancomycin		
A2	22	Stable		-	1					
A2	28	No Sepsis Confirmed		-	1		Flucloxacillin	Gentamicin		
A2	34	Stable		-	1					
A2	41	Stable		-	1					
A2	49	No Sepsis Confirmed		-	1					
A2	56	Stable		-	1					
A2	65	Stable		-	1					
A2	72	Stable		-	1					
A2	79	Stable		-	1					
A20	7	Clinical Sepsis		-	152		Meropenem			
A20	14	Clinical Sepsis		-	18	Excluded	Meropenem			
A20	21	Clinical Sepsis		-	21	Excluded	Meropenem			
A3	4	Microbiology Confirmed Sepsis	*CoNS*	0	1		Ceftazidime	Vancomycin		
A3	10	Microbiology Confirmed Sepsis	*CoNS*	6	1	Excluded				
A3	15	Stable		-	6					
A3	17	Clinical Sepsis		-	1		Flucloxacillin	Gentamicin		
A3	22	Stable		-	1					
A3	30	Stable		-	1					
A3	36	Stable		-	1					
A3	44	Stable		-	1					
A3	51	Stable		-	1					
A3	58	Stable		-	1					
A4	2	No Sepsis Confirmed		-	1		Gentamicin	Benzylpenicillin		
A4	6	Stable		-	1					
A4	8	Microbiology Confirmed Sepsis	*CoNS*	0	26		Vancomycin	Gentamicin		
A5	4	Stable		-	1					
A5	10	Stable		-	1		Gentamicin	Vancomycin		
A5	17	Stable		-	1					
A5	25	Stable		-	1					
A5	26	Stage 2 NEC		-	11	Excluded	Flucloxacillin	Gentamicin		
A5	31	Microbiology Confirmed Sepsis	*CoNS*	0	108		Flucloxacillin	Gentamicin	Metronidazole	

Baby	day of life	sample category	Blood culture	Days from blood culture to research blood draw	CRP on day of research blood draw (mg/L)	Excluded from Fig 1d	Antibiotic 1	Antibiotic 2	Antibiotic 3	Antifungal
A6	8	Microbiology Confirmed Sepsis	*CoNS*	1	1		Ceftazidime	Vancomycin		
A6	22	Stable		-	1					
A6	25	No Sepsis Confirmed		-	11		Flucloxacillin	Gentamicin		
A6	28	Stable		-	1					
A6	35	Stable		-	1					
A7	2	No Sepsis Confirmed		-	1		Gentamicin	Benzylpenicillin		
A7	7	Stable		-	1					
A7	14	Microbiology Confirmed Sepsis	*CoNS*	0	1		Vancomycin			
A7	28	No Sepsis Confirmed		-	1		Flucloxacillin	Gentamicin		
A7	36	No Sepsis Confirmed		-	1		Flucloxacillin	Gentamicin		
A7	42	Stage 2 NEC		-	48	Excluded	Flucloxacillin	Gentamicin	Metronidazole	
A7	49	Stable		-	1					
A7	54	Microbiology Confirmed Sepsis	*GBS*	0	94		Ceftazidime	Vancomycin		
A7	63	Microbiology Confirmed Sepsis	*GBS*	9	1	Excluded	Benzylpenicillin			
A7	70	Stable		-	1					
A7	73	Microbiology Confirmed Sepsis	*GBS*	0	42		Vancomycin	Amikacin		
A8	4	Microbiology Confirmed Sepsis	*E coli*	0	23		Ceftazidime	Vancomycin		
A9	2	Microbiology Confirmed Sepsis	*E coli*	1	21		Gentamicin	Benzylpenicillin	Amoxicillin	
A9	8	Microbiology Confirmed Sepsis	*E coli*	7	1	Excluded	Gentamicin	Benzylpenicillin		
A9	14	Stable		-	7		Ceftazidime			
A9	21	No Sepsis Confirmed		-	1		Meropenem	Vancomycin		
A9	28	Clinical Sepsis		-	25		Flucloxacillin	Gentamicin		
A9	35	Stable		-	1					
A9	38	No Sepsis Confirmed		-	1		Meropenem			
A9	42	Stable		-	1					
A9	49	Stable		-	1					
A9	58	Stable		-	1					
A9	65	Stable		-	1					
A9	70	Stable		-	1					
A9	77	Stable		-	1					
A9	84	Stable		-	1					
B6	6	No Sepsis Confirmed		-	9		Gentamicin	Benzylpenicillin		
B6	11	Clinical Sepsis		-	23		Vancomycin	Ceftazidime		
B6	17	Clinical Sepsis		-	8		Vancomycin	Ceftazidime		
B6	24	Stage 2 NEC		-	14	Excluded	Meropenem	Vancomycin		
B6	30	Stage 2 NEC		-	7	Excluded	Meropenem	Metronidazole		
B6	38	Stage 3 NEC		-	23	Excluded	Meropenem	Vancomycin	Clarithromycin	
B6	45	Stage 3 NEC		-	67	Excluded	Meropenem	Vancomycin	Metronidazole	Amphotericin

GBS = Group B streptococcus

E. coli = Escherichia coli

E.faecalis = Enterococcus faecalis

E.cloacae = Enterobacter cloacae

CoNS = Coagulase negative staphylococcus

Supplementary Table 2. Immune parameters tested by flow cytometry.

Immune parameter	Description
**B cells**	
B cells/ml blood Panel 2A	B cell number
All cells/Single Cells A/Single Cells B/Live cells/B cells | Freq. of Parent of 2A	B cells (%)
All cells/Single Cells A/Single Cells B/Live cells/B cells/CD19+ CD86+ | Freq. of Parent	B cells (%CD86+; activation marker)
**T cells**	
Lymphocytes/Single Cells A/Single Cells B/Live cells/CD3ALL/VD1-VD2-/CD25+ | Freq. of Parent	non Vδ1+ Vδ2+ T cells (% CD25+; activation marker)
Lymphocytes/Single Cells A/Single Cells B/Live cells/CD3ALL/VD1-VD2-/CD95+ | Freq. of Parent	non Vδ1+ Vδ2+ T cells (% CD95/FAS+)
Lymphocytes/Single Cells A/Single Cells B/live cells/T cells | Freq. of Parent	T cells (%)
All cells/Single Cells A/Single Cells B/Live cells/Non B cells/T cells/CD86+ | Freq. of Parent	T cells (%CD86+; activation marker)
All cells/Single Cells A/Single Cells B/Live cells/T cells/HLADR+ T cells | Freq. of Parent	T cells (%HLADR+; activation marker)
Lymphocytes/Single Cells A/Single Cells B/live cells/CD3 all/Ki67+ | Freq. of Parent	Total CD3+ T cells (% Ki67+; marker of proliferation)
**CD4+ T cells**	
Lymphocytes/Single Cells A/Single Cells B/live cells/CD3 all/VD2, VD1 neg/Q1: CD8a- , CD4+/CD4+ CD27+FOXP3+ T reg | Freq. of Parent	CD4+ CD27+FOXP3+ T cells (%)
CD4+ T /ml blood panel 3	CD4+ T cell number
Lymphocytes/Single Cells A/Single Cells B/Live cells/T cells/CD4+/RTE | Freq. of Parent	CD4+ T cell Recent Thymic Emigrants (CD45RA+CD31+)
Lymphocytes/Single Cells A/Single Cells B/live cells/T cells/non gd T cells/non CD3+CD56+/Q1: CD8- , CD4+/T cell CD161+ gate | Freq. of Parent	CD4+ T cells (% CD161+)
Lymphocytes/Single Cells A/Single Cells B/Live cells/CD3ALL/VD1-VD2-/Q1: CD8a- , CD4+/CD25+ | Freq. of Parent	CD4+ T cells (% CD25+; activation marker)
Lymphocytes/Single Cells A/Single Cells B/live cells/T cells/non gd T cells/non CD3+CD56+/Q1: CD8- , CD4+/T cell CD38+ gate | Freq. of Parent	CD4+ T cells (% CD38+)
Lymphocytes/Single Cells A/Single Cells B/live cells/T cells/non gd T cells/non CD3+CD56+/Q1: CD8- , CD4+/T cell CD69+ gate | Freq. of Parent	CD4+ T cells (% CD69+; activation marker)
Lymphocytes/Single Cells A/Single Cells B/Live cells/CD3ALL/VD1-VD2-/Q1: CD8a- , CD4+/CD95+ | Freq. of Parent	CD4+ T cells (% CD95/FAS+)
Lymphocytes/Single Cells A/Single Cells B/Live cells/CD4/CD4 IL-8 | Freq. of Parent	CD4+ T cells (% CXCL8/IL-8+)
Lymphocytes/Single Cells A/Single Cells B/Live cells/CD4/CD4 IFNg | Freq. of Parent	CD4+ T cells (% IFN-γ+)
Lymphocytes/Single Cells A/Single Cells B/Live cells/T cells/CD4+/CD4 IL-10+ | Freq. of Parent	CD4+ T cells (% IL-10+)
Lymphocytes/Single Cells A/Single Cells B/Live cells/CD4/CD4 IL-13 | Freq. of Parent	CD4+ T cells (% IL-13+)
Lymphocytes/Single Cells A/Single Cells B/Live cells/CD4/CD4 IL-17A | Freq. of Parent	CD4+ T cells (% IL-17A/F+)
Lymphocytes/Single Cells A/Single Cells B/Live cells/T cells/CD4+/CD4 IL-2+ | Freq. of Parent	CD4+ T cells (% IL-2+)
Lymphocytes/Single Cells A/Single Cells B/Live cells/T cells/CD4+/CD4 IL-4+ | Freq. of Parent	CD4+ T cells (% IL-4+)
Lymphocytes/Single Cells A/Single Cells B/live cells/CD3 all/VD2, VD1 neg/Q1: CD8a- , CD4+/Ki67+ | Freq. of Parent	CD4+ T cells (% Ki67+; marker of proliferation)
Lymphocytes/Single Cells A/Single Cells B/Live cells/CD3ALL/VD1-VD2-/Q1: CD8a- , CD4+/Q2: CD45RA+ , CCR7+ | Freq. of Parent	CD4+ T cells (% naïve; CD45RA+CCR7+)
Lymphocytes/Single Cells A/Single Cells B/live cells/T cells/non gd T cells/non CD3+CD56+/Q1: CD8- , CD4+/T cell NKG2D+ gate | Freq. of Parent	CD4+ T cells (% NKG2D+)
Lymphocytes/Single Cells A/Single Cells B/Live cells/CD4/CD4 TNFa | Freq. of Parent	CD4+ T cells (% TNFα+)
Lymphocytes/Single Cells A/Single Cells B/live cells/T cells/non gd T cells/non CD3+CD56+/Q1: CD8- , CD4+ | Freq. of Parent	CD4+ T cells (%)
Lymphocytes/Single Cells A/Single Cells B/Live cells/T cells/CD4+/CD35 | Freq. of Parent	CD4+ T cells (CD35/Complement Receptor 1+)
Lymphocytes/Single Cells A/Single Cells B/Live cells/CD3ALL/VD1-VD2-/Q1: CD8a- , CD4+/T reg | Freq. of Parent	CD4+ T regulatory cells (%)
**CD8+ T cells**	
Lymphocytes/Single Cells A/Single Cells B/live cells/CD3 all/VD2, VD1 neg/Q3: CD8a+ , CD4-/CD8+ CD27+FOXP3+ T reg | Freq. of Parent	CD8+ CD27+FOXP3+ T cells (%)
CD8+ T /ml blood panel 3	CD8+ T cell number
Lymphocytes/Single Cells A/Single Cells B/live cells/T cells/non gd T cells/non CD3+CD56+/Q3: CD8+ , CD4-/T cell CD161+ gate | Freq. of Parent	CD8+ T cells (% CD161+)
Lymphocytes/Single Cells A/Single Cells B/Live cells/CD3ALL/VD1-VD2-/Q3: CD8a+ , CD4-/CD25+ | Freq. of Parent	CD8+ T cells (% CD25+; activation marker)
Lymphocytes/Single Cells A/Single Cells B/live cells/T cells/non gd T cells/non CD3+CD56+/Q3: CD8+ , CD4-/T cell CD38+ gate | Freq. of Parent	CD8+ T cells (% CD38+)
Lymphocytes/Single Cells A/Single Cells B/live cells/T cells/non gd T cells/non CD3+CD56+/Q3: CD8+ , CD4-/T cell CD69+ gate | Freq. of Parent	CD8+ T cells (% CD69+; activation marker)
Lymphocytes/Single Cells A/Single Cells B/Live cells/CD3ALL/VD1-VD2-/Q3: CD8a+ , CD4-/CD95+ | Freq. of Parent	CD8+ T cells (% CD95/FAS+)
Lymphocytes/Single Cells A/Single Cells B/Live cells/Non CD4/CD8/CD8 IL-8 | Freq. of Parent	CD8+ T cells (% CXCL8/IL-8+)
Lymphocytes/Single Cells A/Single Cells B/Live cells/Non CD4/CD8/CD8 IFNg | Freq. of Parent	CD8+ T cells (% IFN-γ+)
Lymphocytes/Single Cells A/Single Cells B/Live cells/T cells/CD8+/CD8 IL-10+ | Freq. of Parent	CD8+ T cells (% IL-10+)
Lymphocytes/Single Cells A/Single Cells B/Live cells/Non CD4/CD8/CD8 IL-13 | Freq. of Parent	CD8+ T cells (% IL-13+)
Lymphocytes/Single Cells A/Single Cells B/Live cells/Non CD4/CD8/CD8 IL-17A | Freq. of Parent	CD8+ T cells (% IL-17A/F+)
Lymphocytes/Single Cells A/Single Cells B/Live cells/T cells/CD8+/CD8 IL-2+ | Freq. of Parent	CD8+ T cells (% IL-2+)
Lymphocytes/Single Cells A/Single Cells B/Live cells/T cells/CD8+/CD8 IL-4+ | Freq. of Parent	CD8+ T cells (% IL-4+)
Lymphocytes/Single Cells A/Single Cells B/live cells/CD3 all/VD2, VD1 neg/Q3: CD8a+ , CD4-/Ki67+ | Freq. of Parent	CD8+ T cells (% Ki67+; marker of proliferation)
Lymphocytes/Single Cells A/Single Cells B/Live cells/CD3ALL/VD1-VD2-/Q3: CD8a+ , CD4-/Q2: CD45RA+ , CCR7+ | Freq. of Parent	CD8+ T cells (% naïve; CD45RA+CCR7+)
Lymphocytes/Single Cells A/Single Cells B/live cells/T cells/non gd T cells/non CD3+CD56+/Q3: CD8+ , CD4-/T cell NKG2D+ gate | Freq. of Parent	CD8+ T cells (% NKG2D+)
Lymphocytes/Single Cells A/Single Cells B/Live cells/Non CD4/CD8/CD8 TNFa | Freq. of Parent	CD8+ T cells (% TNFα+)
Lymphocytes/Single Cells A/Single Cells B/live cells/T cells/non gd T cells/non CD3+CD56+/Q3: CD8+ , CD4- | Freq. of Parent	CD8+ T cells (%)
Lymphocytes/Single Cells A/Single Cells B/Live cells/T cells/CD8+/CD35 | Freq. of Parent	CD8+ T cells (CD35/Complement Receptor 1+)
Lymphocytes/Single Cells A/Single Cells B/Live cells/CD3ALL/VD1-VD2-/Q3: CD8a+ , CD4-/T reg | Freq. of Parent	CD8+ T regulatory cells (%)

**gd T cells**	
gd T cells/ml blood panel 3	γδ T cell number
Lymphocytes/Single Cells A/Single Cells B/live cells/T cells/gd T cells/T cell CD161+ gate | Freq. of Parent	γδ T cells (% CD161+)
Lymphocytes/Single Cells A/Single Cells B/live cells/T cells/gd T cells/T cell CD38+ gate | Freq. of Parent	γδ T cells (% CD38+)
Lymphocytes/Single Cells A/Single Cells B/live cells/T cells/gd T cells/T cell CD69+ gate | Freq. of Parent	γδ T cells (% CD69+; activation marker)
Lymphocytes/Single Cells A/Single Cells B/Live cells/Non CD4/Non CD4 pan gd/gd IL-8. | Freq. of Parent	γδ T cells (% CXCL8/IL-8+)
Lymphocytes/Single Cells A/Single Cells B/Live cells/Non CD4/Non CD4 pan gd/gd IFNg. | Freq. of Parent	γδ T cells (% IFN-γ+)
Lymphocytes/Single Cells A/Single Cells B/Live cells/Non CD4/Non CD4 pan gd/gd IL-13. | Freq. of Parent	γδ T cells (% IL-13+)
Lymphocytes/Single Cells A/Single Cells B/Live cells/Non CD4/Non CD4 pan gd/gd IL-17A. | Freq. of Parent	γδ T cells (% IL-17A/F+)
Lymphocytes/Single Cells A/Single Cells B/live cells/T cells/gd T cells/T cell NKG2D+ gate | Freq. of Parent	γδ T cells (% NKG2D+)
Lymphocytes/Single Cells A/Single Cells B/Live cells/Non CD4/Non CD4 pan gd/gd TNFa. | Freq. of Parent	γδ T cells (% TNFα+)
Lymphocytes/Single Cells A/Single Cells B/live cells/T cells/gd T cells | Freq. of Parent	γδ T cells (%)
Lymphocytes/Single Cells A/Single Cells B/Live cells/CD3ALL/VD1/CD25+ | Freq. of Parent	Vδ1 γδ T cell (% CD25+; activation marker)
Lymphocytes/Single Cells A/Single Cells B/Live cells/CD3ALL/VD1/CD95+ | Freq. of Parent	Vδ1 γδ T cell (% CD95/FAS+)
Lymphocytes/Single Cells A/Single Cells B/live cells/CD3 all/VD1/Ki67+ | Freq. of Parent	Vδ1 γδ T cell (% Ki67+; marker of proliferation)
Lymphocytes/Single Cells A/Single Cells B/live cells/CD3 all/VD1/Q2: CD45RA+ , CCR7+ | Freq. of Parent	Vδ1 γδ T cell (% naïve; CD45RA+CCR7+)
Lymphocytes/Single Cells A/Single Cells B/Live cells/CD3ALL/VD1 | Freq. of Parent	Vδ1 γδ T cell (%)
Lymphocytes/Single Cells A/Single Cells B/Live cells/CD3ALL/VD2/CD25+ | Freq. of Parent	Vδ2 γδ T cell (% CD25+; activation marker)
Lymphocytes/Single Cells A/Single Cells B/Live cells/CD3ALL/VD2/CD95+ | Freq. of Parent	Vδ2 γδ T cell (% CD95/FAS+)
Lymphocytes/Single Cells A/Single Cells B/live cells/CD3 all/VD2/Ki67+ | Freq. of Parent	Vδ2 γδ T cell (% Ki67+; marker of proliferation)
Lymphocytes/Single Cells A/Single Cells B/live cells/CD3 all/VD2/Q2: CD45RA+ , CCR7+ | Freq. of Parent	Vδ2 γδ T cell (% naïve; CD45RA+CCR7+)
Lymphocytes/Single Cells A/Single Cells B/Live cells/CD3ALL/VD2 | Freq. of Parent	Vδ2 γδ T cell (%)
**NK cells**	
NK cells/ml blood panel 3	NK cell number
Lymphocytes/Single Cells A/Single Cells B/live cells/Non T cells/NK cells/CD161 NK | Freq. of Parent	NK cells (% CD161+)
Lymphocytes/Single Cells A/Single Cells B/live cells/Non T cells/NK cells/CD38+ NK | Freq. of Parent	NK cells (% CD38+)
Lymphocytes/Single Cells A/Single Cells B/live cells/Non T cells/NK cells/CD69+ NK | Freq. of Parent	NK cells (% CD69+; activation marker)
Lymphocytes/Single Cells A/Single Cells B/Live cells/Non T/NK cells./NK IL-8 | Freq. of Parent	NK cells (% CXCL8/IL-8+)
Lymphocytes/Single Cells A/Single Cells B/Live cells/Non T/NK cells./NK IFNg | Freq. of Parent	NK cells (% IFN-γ+)
Lymphocytes/Single Cells A/Single Cells B/Live cells/Non T/NK cells./NK IL-13 | Freq. of Parent	NK cells (% IL-13+)
Lymphocytes/Single Cells A/Single Cells B/Live cells/Non T/NK cells./NK IL-17A | Freq. of Parent	NK cells (% IL-17A/F+)
Lymphocytes/Single Cells A/Single Cells B/live cells/Non T cells/NK cells/NKG2D NK | Freq. of Parent	NK cells (% NKG2D+)
Lymphocytes/Single Cells A/Single Cells B/Live cells/Non T/NK cells./NK TNFa | Freq. of Parent	NK cells (% TNFα+)
Lymphocytes/Single Cells A/Single Cells B/live cells/Non T cells/NK cells | Freq. of Parent	NK cells (%)
**NKT cells**	
All cells/Single Cells A/Single Cells B/Live cells/T cells/CD3+CD56+ NKT/NKT HLA-DR | Freq. of Parent	NKT cell (%HLADR+; activation marker)
CD3+CD56+/ml blood panel 3	NKT cell number
Lymphocytes/Single Cells A/Single Cells B/live cells/T cells/non gd T cells/CD3+CD56+/T cell CD161+ gate | Freq. of Parent	NKT cells (% CD161+)
Lymphocytes/Single Cells A/Single Cells B/live cells/T cells/non gd T cells/CD3+CD56+/T cell CD38+ gate | Freq. of Parent	NKT cells (% CD38+)
Lymphocytes/Single Cells A/Single Cells B/live cells/T cells/non gd T cells/CD3+CD56+/T cell CD69+ gate | Freq. of Parent	NKT cells (% CD69+; activation marker)
Lymphocytes/Single Cells A/Single Cells B/live cells/T cells/non gd T cells/CD3+CD56+/T cell NKG2D+ gate | Freq. of Parent	NKT cells (% NKG2D+)
Lymphocytes/Single Cells A/Single Cells B/live cells/T cells/non gd T cells/CD3+CD56+ | Freq. of Parent	NKT cells (%)
**Myeloid cells**	
All cells/Single Cells A/Single Cells B/Live cells/HLADR+ cells/CD56 neg /Non T cell/CD86+ cells/Patrolling monocytes | Freq. of Parent	Patrolling monocytes (%)
All cells/Single Cells A/Single Cells B/Live cells/HLADR+ cells/CD56 neg /Non T cell/CD86+ cells/Patrolling monocytes | Median (HLA-DR)	Patrolling monocytes median HLA-DR
All cells/Single Cells A/Single Cells B/Live cells/Non B cells/Non T cells/Dendritic Cells/pDC | Freq. of Parent	plasmacytoid DC (%)
pDC/ml blood Panel 2B	plasmacytoid DC number
All cells/Single Cells A/Single Cells B/Live cells/HLADR+ cells/CD56 neg /Non T cell/CD86+ cells/classical monocytes | Median (HLA-DR)	Classical monocytes - median HLA-DR
All cells/Single Cells A/Single Cells B/Live cells/HLADR+ cells/CD56 neg /Non T cell/CD86+ cells/classical monocytes | Freq. of Parent	Classical monocytes (%)
All cells/Single Cells A/Single Cells B/Live cells/HLADR+ cells/CD56 neg /Non T cell/CD86+ cells/Dc | Freq. of Parent	DC (%)
All cells/Single Cells A/Single Cells B/Live cells/HLADR+ cells/CD56 neg /Non T cell/CD86+ cells/Dc | Median (HLA-DR)	DC median HLA-DR
All cells/Single Cells A/Single Cells B/Live cells/HLADR+ cells/CD56 neg /Non T cell/CD86+ cells/Int monocytes | Freq. of Parent	Intermediate monocytes (%)
All cells/Single Cells A/Single Cells B/Live cells/HLADR+ cells/CD56 neg /Non T cell/CD86+ cells/Int monocytes | Median (HLA-DR)	Intermediate monocytes median HLA-DR
All cells/Single Cells A/Single Cells B/Live cells/Non B cells/Non T cells/monocytes | Freq. of Parent	monocytes (%)
monocytes/ml blood Panel 2B	monocytes number
All cells/Single Cells A/Single Cells B/Live cells/Non B cells/Non T cells/Dendritic Cells/mDC | Freq. of Parent	myeloid DC (%)
mDC/ml blood Panel 2B	myeloid DC number

Supplementary Table 3. Antibody panels for flow cytometry.

Panel 1: T cell phenotype					
Marker	Fluor	Company	Cat No.	Clone	Dilution
CD3	AF700	Biolegend	317339	OKT3	1 in 200
CD4	PE-Cy7	Biolegend	317413	OKt4	1 in 50
CD8	BV510	Biolegend	301047	RPA-T8	1 in 400
Vδ1	FITC	Thermo Scientific	TCR2730	TS8.2	1 in 100
Vδ2	PerCP	Biolegend	331410	B6	1 in 50
CD45RA	BV786	Biolegend	304139	HI 100	1 in 50
CCR7	BV650	Biolegend	353233	GO43H7	1 in 50
CD95	BV421	Biolegend	305623	dx2	1 in 50
CD25	PE	Biolegend	356103	MA-251	1 in 50
FoxP3	APC	Biolegend	320213	259 d	1 in 50
Human Trustain FcX	Fc Block	Biolegend	422302	-	2.5 in 50

Panel 2: T cell phenotype					
Marker	Fluor	Company	Cat No.	Clone	Dilution
CD3	AF700	Biolegend	317339	OKT3	1 in 200
CD4	PE-Cy7	Biolegend	317413	OKt4	1 in 50
CD8	BV510	Biolegend	301047	RPA-T8	1 in 400
Vδ1	FITC	Thermo Scientific	TCR2730	TS8.2	1 in 100
Vδ2	PerCP	Biolegend	331410	B6	1 in 50
CD45RA	BV786	Biolegend	304139	HI 100	1 in 50
CCR7	BV650	Biolegend	353233	GO43H7	1 in 50
Ki67	BV421	Biolegend	350505	Ki-67	1 in 50
CD27	PE	Biolegend	356103	MA-251	1 in 50
FoxP3	APC	Biolegend	320214	259 d	1 in 50
Human Trustain FcX	Fc Block	Biolegend	422302	-	2.5 in 50

Panel 3: T/NK/γδ T cell phenotype					
Marker	Fluor	Company	Cat No.	Clone	Dilution
CD3	Af700	Biolegend	317339	OKT3	1 in 200
TCR-γδ	PE-Cy7	Biolegend	331222	B1	1 in 50
CD4	BV786	Biolegend	317442	Okt4	1 in 50
CD8	BV510	Biolegend	301047	RPA-T8	1 in 400
NKG2D	APC	Biolegend	320807	1D11	1 in 50
CD56	BV421	Biolegend	362551	5.1h11	1 in 200
CD69	PerCP-Cy5.5	Biolegend	310925	FN50	1 in 50
CD161	FITC	Biolegend	339923	HP-3G10	1 in 50
CD38	BV650	Biolegend	356619	HB-7	1 in 100
CD16	APC CY7	Biolegend	302018	3G8	1 in 100
CD14	APC CY7	Biolegend	325620	HCD14	1 in 100
Human Trustain FcX	Fc Block	Biolegend	422302	-	2.5 in 50

Panel 4: T/NK/$\gamma\delta$ T cell function					
Marker	Fluor	Company	Cat No.	Clone	Dilution
CD3	AF700	Biolegend	317339	OKT3	1 in 200
TCR-$\gamma\delta$	PE-Cy7	Biolegend	331222	B1	1 in 50
CD4	BV786	Biolegend	317442	Okt4	1 in 50
CD8	BV510	Biolegend	301047	RPA-T8	1 in 400
CD56	BV650	Biolegend	362532	5.1h11	1 in 50
Human Trustain FcX	Fc Block	Biolegend	422302	-	2.5 in 50
IFNγ	BV421	Biolegend	502531	4S.B3	1 in 50
TNFα	PerCP-Cy5.5	Biolegend	502925	MAB11	1 in 50
IL17A	AF647	Biolegend	512309	BL168	1 in 50
IL17F	AF647	Biolegend	517003	9D3.1C8	1 in 50
IL-13	PE	Biolegend	501903	JES10-5A2	1 in 50
IL-8	FITC	Biolegend	511406	E8N1	1 in 50

Panel 5: T cell function					
Marker	Fluor	Company	Cat No.	Clone	Dilution
CD3	AF700	Biolegend	317339	OKT3	1 in 200
CD4	BV786	Biolegend	317442	Okt4	1 in 50
CD8	BV510	Biolegend	301047	RPA-T8	1 in 400
CD45RA	BV650	Biolegend	304135	HI100	1 in 100
CD31	PerCP-Cy5.5	Biolegend	303131	WM59	1 in 400
CD35	AF647	BD	565329	E11	1 in 50
Human Trustain FcX	Fc Block	Biolegend	422302	-	2.5 in 50
IL-8	FITC	Biolegend	511406	E8N1	1 in 50
IL-2	PE-CY7	Biolegend	500325	MQ1-17H12	1 in 50
IL-4	PE	Biolegend	500808	MP425d2	1 in 50
IL-10	BV421	Biolegend	501421	Jes3-97d	1 in 50

Panel 6: Monocyte panel					
Marker	Fluor	Company	Cat No.	Clone	Dilution
CD3	AF700	Biolegend	317339	OKt3	1 in 200
CD19	BV786	Biolegend	302239	HIB 19	1 in 100
CD14	BV421	Biolegend	301829	M5E2	1 in 100
CD16	PE-Cy7	Biolegend	302015	3g8	1 in 200
HLADR	FITC	Biolegend	307619	L243	1 in 100
CD86	PE	Biolegend	305405	IT2.2	1 in 100
CD40	BV510	Biolegend	334329	5c3	1 in 100
CD1c	APC	Biolegend	331523	L161	1 in 100
CD56	BV650	Biolegend	362532	5.1h11	1 in 50
CD123	PerCP Cy5.5	Biolegend	306015	6H6	1 in 50
Human Trustain FcX	Fc Block	Biolegend	422302	-	2.5 in 50

Panel 7: Dendritic cell panel					
Marker	Fluor	Company	Cat No.	Clone	Dilution
CD3	AF700	Biolegend	317339	OKt3	1 in 200
CD19	BV786	Biolegend	302239	HIB 19	1 in 100
CD14	BV421	Biolegend	301829	M5E2	1 in 100
CD16	PE-Cy7	Biolegend	302015	3g8	1 in 200
CD303	FITC	Biolegend	307619	L243	1 in 100
CD86	PE	Biolegend	305405	IT2.2	1 in 100
CD40	BV510	Biolegend	334329	5c3	1 in 100
CD1c	APC	Biolegend	331523	L161	1 in 100
CD11c	BV650	Biolegend	301637	3.9	1 in 50
CD123	PerCP Cy5.5	Biolegend	306015	6H6	1 in 50
Human Trustain FcX	Fc Block	Biolegend	422302	-	2.5 in 50

Limited functional panel for samples used in scRNA-seq analysis (babies A5, A11, A12, B6 only)					
Marker	Fluor	Company	Cat No.	Clone	Dilution
CD4	BV786	Biolegend	317442	Okt4	1 in 50
IFNγ	BV650	Biolegend	502538	4S.B3	1 in 50
CD8	BV605	Biolegend	301040	RPA-T8	1 in 100
IL-2	BV510	Biolegend	500338	MQ1-17H12	1 in 50
CD56	BV421	Biolegend	362551	5.1h11	1 in 200
TCR-$\gamma\delta$	PE-Cy7	Biolegend	331222	B1	1 in 50
IL-10	PE Dazzle	Biolegend	501426	JES3-9D7	1 in 50
IL-4	PE	Biolegend	500705	8D4-8	1 in 50
TNFα	PErCP Cy5.5	Biolegend	502926	MAb11	1 in 50
IL-8	FITC	Biolegend	511406	E8N1	1 in 50
CD3	AF700	Biolegend	317339	OKT3	1 in 200
IL17A	AF647	Biolegend	512310	BL168	1 in 50
IL17F	AF647	BD	561333	033-782	1 in 50
Human Trustain FcX	Fc Block	Biolegend	422302	-	2.5 in 50

Panel 8: Amphiregulin					
Marker	Fluor	Company	Cat No.	Clone	Dilution
CD4	BV786	Biolegend	317442	Okt4	1 in 50
CD34	BV650	Biolegend	343608	561	1 in 50
CD8	BV510	Biolegend	301047	RPA-T8	1 in 400
CD56	BV421	Biolegend	362551	5.1h11	1 in 200
TCR-$\gamma\delta$	PE-Cy7	Biolegend	331222	B1	1 in 50
CD19	PE Dazzle 594	Biolegend	302252	HIB19	1 in 100
CD14	PerCP Cy5.5	Biolegend	367110	63D3	1 in 50
CD3	AF700	Biolegend	317339	OKT3	1 in 200
Amphiregulin	APC	eBioscience	17-5370-42	AREG559	1 in 50
IL-8	FITC	Biolegend	511406	E8N1	1 in 50
IFNγ	PE	Biolegend	502509	4S.B3	1 in 50
IL-17	BV605	Biolegend	512326	BL168	1 in 50
Human Trustain FcX	Fc Block	Biolegend	422302	-	2.5 in 50
