# Supplementary material for: Identifying immune signatures of sepsis to increase diagnostic accuracy in very preterm babies
Source: Nat Commun. 2024 Jan 9;15:388. doi: 10.1038/s41467-023-44387-5 (PMC10776581; doi:10.1038/s41467-023-44387-5)
Supplement: Supplementary file 3 — Reporting Summary [file 41467_2023_44387_MOESM3_ESM.pdf]

nature portfolio

Corresponding author(s): Abhishek Das, Deena Gibbons

Last updated by author(s): Nov 22, 2023

Reporting Summary

Nature Portfolio wishes to improve the reproducibility of the work that we publish. This form provides structure for consistency and transparency in reporting. For further information on Nature Portfolio policies, see our Editorial Policies and the Editorial Policy Checklist.

Statistics

For all statistical analyses, confirm that the following items are present in the figure legend, table legend, main text, or Methods section.

n/a	Confirmed	
☐	☒	The exact sample size (*n*) for each experimental group/condition, given as a discrete number and unit of measurement
☐	☒	A statement on whether measurements were taken from distinct samples or whether the same sample was measured repeatedly
☐	☒	The statistical test(s) used AND whether they are one- or two-sided<br>*Only common tests should be described solely by name; describe more complex techniques in the Methods section.*
☐	☒	A description of all covariates tested
☐	☒	A description of any assumptions or corrections, such as tests of normality and adjustment for multiple comparisons
☐	☒	A full description of the statistical parameters including central tendency (e.g. means) or other basic estimates (e.g. regression coefficient) AND variation (e.g. standard deviation) or associated estimates of uncertainty (e.g. confidence intervals)
☐	☒	For null hypothesis testing, the test statistic (e.g. *F*, *t*, *r*) with confidence intervals, effect sizes, degrees of freedom and *P* value noted<br>*Give P values as exact values whenever suitable.*
☒	☐	For Bayesian analysis, information on the choice of priors and Markov chain Monte Carlo settings
☒	☐	For hierarchical and complex designs, identification of the appropriate level for tests and full reporting of outcomes
☒	☐	Estimates of effect sizes (e.g. Cohen's *d*, Pearson's *r*), indicating how they were calculated

Our web collection on statistics for biologists contains articles on many of the points above.

Software and code

Policy information about availability of computer code

Data collection	Clinical, microbiological and laboratory data: extracted from the Badgernet database (patient electronic records). Flow cytometry data collection: BD FACSDiva Software v8.0.1. scRNAseq data: Cellranger v5.0.1(10X genomics).
Data analysis	-FlowJo v10.6.2 (BD)<br>-Figures and statistical analyses for flow cytometry experiments were generated using JMP Pro v17.<br>-Diagrams (Fig. 1a-b, Fig. 2a, Supplementary Figure 2a, Supplementary Figure 3 and Supplementary Figure 15) were generated on BioRender.com.<br>-Single cell data were processed in R (v.4.0.2) usingSeurat (v4.0.1), scater (v1.18.6) and harmony (v1.0 immunogenics lab) packages. The singleR package was used to annotate cells within the scRNA-seq dataset against the Monaco reference transcriptomic dataset. Major immune cell clusters were further manually curated by expression of canonical marker genes using Loupe Browser (v5.1.0; 10X Genomics). Assessment of differentially expressed genes used the differential expression algorithm in Loupe Browser (v5.1.0; 10X Genomics), which is based on the negative binomial exact test used in the sSeq method.<br>Data were then exported as .csv files into JMP Pro v17 to create volcano plots.<br>-Venn diagrams were created using the web-based tool DiVenn 1.2 (https://divenn.tch.harvard.edu/).<br>-The Forest plot in Figure. 4c was generated using the web-based tool MetaSignature; available here: https://metasignature.stanford.edu/<br>-Gene set enrichment analysis was performed using the web-based tool Enrich-KG (https://maayanlab.cloud/enrichr-kg).<br>-Multiplex bead array data were analysed using Windows LegendPlex software (v8.0, BioLegend).

For manuscripts utilizing custom algorithms or software that are central to the research but not yet described in published literature, software must be made available to editors and reviewers. We strongly encourage code deposition in a community repository (e.g. GitHub). See the Nature Portfolio guidelines for submitting code & software for further information.

Data

Policy information about availability of data

All manuscripts must include a data availability statement. This statement should provide the following information, where applicable:

- Accession codes, unique identifiers, or web links for publicly available datasets
- A description of any restrictions on data availability
- For clinical datasets or third party data, please ensure that the statement adheres to our policy

The flow cytometry data (FCS files) generated in this study have been deposited in the flowrepository database under accession code FR-FCM-Z6LE (http://flowrepository.org/id/FR-FCM-Z6LE). Our current dataset incorporates FCS files obtained from seven babies who were included in a parent study derived from the same patient cohort; Kamdar et al. (https://doi.org/10.1038/s41467-020-14923-8). For these babies, the FCS files were downloaded from http://flowrepository.org/id/FR-FCM-Z2FJ. As FCS files cannot be kept under two ID numbers on flowrepository.org, data from these 7 babies will remain under the ID: FR-FCM-Z2FJ. A list of Baby ID conversions are provided here, in order to locate the samples on flowrepository: Baby A1=5006; Baby A2=5040; Baby A3=5051; Baby A4=5060; Baby A7=5025; Baby A9=5018 and Baby A10=5059.
Public datasets used in the present study: Meta-analysis of public gene expression microarrays to assess cell specific expression of amphiregulin was carried out using the online MetaSignature tool developed by the Khatri Lab (https://metasignature.stanford.edu/). A Forest plot was created and downloaded from the web-based tool.
The scRNA-seq data generated in this study have been deposited in the NCBI Gene Expression Omnibus repository under accession code GSE236099 (https://www.ncbi.nlm.nih.gov/geo/query/acc.cgi?acc=GSE236099). Source data are provided with this paper.

Research involving human participants, their data, or biological material

Policy information about studies with human participants or human data. See also policy information about sex, gender (identity/presentation), and sexual orientation and race, ethnicity and racism.

Reporting on sex and gender	Data on the sex of a baby was collected directly from the clinical electronic notes as documented. We did not select babies based on sex; rather all preterm babies born before 32 weeks gestation and who encountered one or more episodes of sepsis or necrotising enterocolitis (NEC) were included.<br>Sex for each baby is shown in Table 1.<br>There was no predominance of sepsis in either males or females within the cohort.<br>Flow cytometry cohort: % Female= 47.4% (9/19 babies).<br>Plasma amphiregulin/cytokines: % Female = 48% (11/23 babies).<br>ScRNAseq: % Female = 60% (3/5 babies).<br>Sepsis-induced perturbations in several immune traits including amphiregulin were preserved after adjustment for sex, as well as other confounders, within our multivariate analysis.
Reporting on race, ethnicity, or other socially relevant groupings	Ethnicity was not included in the manuscript in an effort to limit the number of indirect identifiers so that participants remain anonymous.<br>Confounding variables (including sex, postnatal age, gestational age and others) were controlled for via multivariate analysis or sub-grouping samples prior to analysis e.g. given that postnatal age is known to influence immunity, samples were first subgrouped into <30 days and >30 days age brackets prior to assessing the influence of sepsis on immunity.
Population characteristics	Flow cytometry analysis: 19 preterm babies with a median gestational age of 24 weeks and median birthweight of 670 grams. All babies included in the flow cytometry study had one or more episodes of sepsis or NEC. A median of 9 blood draws were taken from each baby at approximately weekly intervals. From a total of 157 blood samples, 47 represented samples taken during sepsis and 6 when a baby was confirmed to have NEC. The remainder represented samples obtained when either a) sepsis was not confirmed and antibiotics were stopped within 72 hours or b) there was no evidence of infection.
Recruitment	Preterm babies (born before 32 weeks gestation), admitted to the neonatal unit before 72 hours of age were recruited to the study.<br>Informed consent was obtained from parents for all babies recruited.<br>Recruitment occurred between January 2016 and August 2018 at a single neonatal intensive care unit in London.<br>Babies with known exposure to HIV or hepatitis B virus were excluded.<br>A translator was present if English was not the parent's first language.<br>Blood was taken at weekly intervals or when sepsis or NEC were suspected. PBMC were extracted and stored in liquid nitrogen.<br>Flow cytometry analysis: The aim of our present study was to understand the influence of bacterial sepsis or NEC on host immune dysregulation in preterm babies. To address this, clinical colleagues identified a sub-cohort of babies (for whom we had PBMC frozen) who were subsequently proven to have microbiological confirmed infection, NEC or 'clinical sepsis' to be included in the study. The immunology team, who ran the experiments, were therefore aware that recruited babies had encountered sepsis or NEC, however were blinded as to which of the PBMC samples represented the clinical event timepoint (median 9 samples per baby). Sample-level categorisation was only assigned after flow cytometry evaluation, by two neonatology consultants.<br>Single-cell RNA seq analysis: our aim was to assess differential gene expression within PBMC between a sepsis or NEC sample

	versus the temporally closest blood sample available from the same baby. Therefore, samples selected specifically for scRNA-seq were selected by the clinical team, and unblinded to the immunology team.<br><br>Parents for all babies born before 32 weeks gestation were approached for informed consent over two years. Additionally, translators were made available when required. Therefore, we do not envisage any significant self-selection bias.
Ethics oversight	Homerton Hospital Cohort: Ethical approval was granted by the London (Chelsea) Research Ethics Committee (Ref: 15/LO/1924) and the Research and Innovation Development at Homerton Healthcare NHS Foundation Trust.<br>St Thomas' Hospital Cohort: Ethical approval granted by the London - Fulham Research Ethics Committee, Ref: 20/PR/0964<br>Cord blood samples were purchased from the Anthony Nolan Research Tissue Biobank (Cell and Gene Therapy Services) after informed consent [Ethical approval NRES Committee East Midlands - Derby Ref 15/EM/0045]. Control adult blood samples were collected after informed consent [Ethical approval granted by the London - Fulham Research Ethics Committee, Ref: 20/PR/0964].

Note that full information on the approval of the study protocol must also be provided in the manuscript.

Field-specific reporting

Please select the one below that is the best fit for your research. If you are not sure, read the appropriate sections before making your selection.

☒ Life sciences ☐ Behavioural & social sciences ☐ Ecological, evolutionary & environmental sciences

For a reference copy of the document with all sections, see nature.com/documents/nr-reporting-summary-flat.pdf

Life sciences study design

All studies must disclose on these points even when the disclosure is negative.

Sample size	Given that preterm physiology and immunological development rapidly evolves over a few short weeks, we restricted our analysis to babies less than 32 weeks gestation (median gestational age 24 weeks). No sample size calculation was carried out given our cohort was restricted to babies at the very earliest gestation. Instead, babies were sampled longitudinally to remove confounders including inter-individual heterogeneity and to control for other factors including age.
Data exclusions	Flow cytometry: After flow cytometry gating, cell populations were excluded from downstream analysis if the event count in the parent population did not meet a minimum threshold of 30 events. A comment is added to the figure legends which states that n numbers may vary slightly between graphs due to this additional quality control step.<br>Given that blood samples were obtained sequentially at weekly intervals, in some circumstances blood was collected more than once during the same sepsis episode. In such cases, only the first sample was used for analysis and subsequent samples from the same sepsis episode were ignored. This is clearly depicted in figure 1c.<br>ScRNA-seq: Haemaglobin genes, which likely represented blood contamination, were filtered out prior to analysis of differential gene expression within PBMC during the scRNA-seq analysis.<br>Plasma analysis: Given that blood samples were obtained sequentially at weekly intervals, in some circumstances, blood was collected more than once during the same sepsis episode. In such cases, only the first sample was used for analysis and subsequent samples from the same sepsis episode were ignored. This is clearly depicted in figure 6b.
Replication	Due to the small volume of blood obtained (0.5ml) from mainly extremely preterm babies, replicates whilst doing the flow cytometry examples were not performed. This was also the case for scRNA-seq and plasma analysis (50-100 microlitres of plasma was available precluding technical replicates).
Randomization	Babies were not randomised in this study. As part of a larger prospective observational study running between 2016 and 2018, preterm babies (less than 32 weeks gestation) on the neonatal unit, had blood samples taken at weekly intervals, and additionally if sepsis or necrotising enterocolitis were suspected. PBMC were extracted and stored in liquid nitrogen. Similarly, babies recruited from St Thomas' hospital, included in our plasma amphiregulin cohort, were sampled prospectively, therefore it was not known which babies were likely to develop sepsis.<br><br>Given that our aim was to study longitudinal changes in immune phenotype associated with sepsis or NEC, our clinical team specifically identified a sub-cohort of babies who were proven to have sepsis or NEC. Frozen PBMC from these babies were thawed and analysed by flow cytometry and scRNA-seq.
Blinding	Blinding of patient recruitment was not possible given that our study specifically recruited babies who had encountered confirmed sepsis for flow cytometry immunophenotyping. Though the immunology team who ran the experiments knew that babies had been specificially identified based on proven sepsis, they were nevertheless blinded to which of the individual samples from that baby (median 9 samples / baby) represented the index clinical event. Exception to this rule: For the five babies in whom scRNA-seq was performed; both the clinical and immunology team were unblinded to sample level data such that the most clinically relevant samples could be interrogated by scRNA-seq.

Reporting for specific materials, systems and methods

We require information from authors about some types of materials, experimental systems and methods used in many studies. Here, indicate whether each material, system or method listed is relevant to your study. If you are not sure if a list item applies to your research, read the appropriate section before selecting a response.

Materials & experimental systems

n/a	Involved in the study
☐	☒ Antibodies
☒	☐ Eukaryotic cell lines
☒	☐ Palaeontology and archaeology
☒	☐ Animals and other organisms
☐	☒ Clinical data
☒	☐ Dual use research of concern
☒	☐ Plants

Methods

n/a	Involved in the study
☒	☐ ChIP-seq
☐	☒ Flow cytometry
☒	☐ MRI-based neuroimaging

Antibodies

Antibodies used

All flow cytometry antibodies used in the study are itemized in Supplementary Table 3 and are additionally shown below. All Totalseq-B or Totalseq-C antibodies and hashtags used for Cite-seq are shown in a table in Supplementary Figure 7 (and additionally below).
Panel 1: T cell phenotype
Marker / Fluor / Company / Cat No. /Clone / Dilution
CD3 AF700 Biolegend 317339 OKT3 1 in 200
CD4 PE-Cy7 Biolegend 317413 OKt4 1 in 50
CD8 BV510 Biolegend 301047 RPA-T8 1 in 400
Vd1 FITC Thermo Scientific TCR2730 TS8.2 1 in 100
Vd2 PerCP Biolegend 331410 B6 1 in 50
CD45RA BV786 Biolegend 304139 HI 100 1 in 50
CCR7 BV650 Biolegend 353233 GO43H7 1 in 50
CD95 BV421 Biolegend 305623 dx2 1 in 50
CD25 PE Biolegend 356103 MA-251 1 in 50
FoxP3 APC Biolegend 320213 259 d 1 in 50
Human Trustain FcX Fc Block Biolegend 422302 - 2.5 in 50

Panel 2: T cell phenotype
Marker Fluor Company Cat No. Clone Dilution
CD3 AF700 Biolegend 317339 OKT3 1 in 200
CD4 PE-Cy7 Biolegend 317413 OKt4 1 in 50
CD8 BV510 Biolegend 301047 RPA-T8 1 in 400
Vd1 FITC Thermo Scientific TCR2730 TS8.2 1 in 100
Vd2 PerCP Biolegend 331410 B6 1 in 50
CD45RA BV786 Biolegend 304139 HI 100 1 in 50
CCR7 BV650 Biolegend 353233 GO43H7 1 in 50
Ki67 BV421 Biolegend 350505 Ki-67 1 in 50
CD27 PE Biolegend 356103 MA-251 1 in 50
FoxP3 APC Biolegend 320214 259 d 1 in 50
Human Trustain FcX Fc Block Biolegend 422302 - 2.5 in 50

Panel 3: T/NK/TCRgd phenotype
Marker Fluor Company Cat No. Clone Dilution
CD3 Af700 Biolegend 317339 OKT3 1 in 200
TCRgd PE-Cy7 Biolegend 331222 B1 1 in 50
CD4 BV786 Biolegend 317442 Okt4 1 in 50
CD8 BV510 Biolegend 301047 RPA-T8 1 in 400
NKG2D APC Biolegend 320807 1D11 1 in 50
CD56 BV421 Biolegend 362551 5.1h11 1 in 200
CD69 PerCP-Cy5.5 Biolegend 310925 FN50 1 in 50
CD161 FITC Biolegend 339923 HP-3G10 1 in 50
CD38 BV650 Biolegend 356619 HB-7 1 in 100
CD16 APC CY7 Biolegend 302018 3G8 1 in 100
CD14 APC CY7 Biolegend 325620 HCD14 1 in 100
Human Trustain FcX Fc Block Biolegend 422302 - 2.5 in 50

Panel 4: T/NK/TCRgd function
Marker Fluor Company Cat No. Clone Dilution
CD3 AF700 Biolegend 317339 OKT3 1 in 200
TCRgd PE-Cy7 Biolegend 331222 B1 1 in 50

CD4 BV786 Biolegend 317442 Okt4 1 in 50
CD8 BV510 Biolegend 301047 RPA-T8 1 in 400
CD56 BV650 Biolegend 362532 5.1h11 1 in 50
Human Trustain FcX Fc Block Biolegend 422302 - 2.5 in 50
IFNg BV421 Biolegend 502531 4S.B3 1 in 50
TNFa PerCP-Cy5.5 Biolegend 502925 MAB11 1 in 50
IL17A AF647 Biolegend 512309 BL168 1 in 50
IL17F AF647 Biolegend 517003 9D3.1C8 1 in 50
IL-13 PE Biolegend 501903 JES10-5A2 1 in 50
IL-8 FITC Biolegend 511406 E8N1 1 in 50

Panel 5: T cell function
Marker Fluor Company Cat No. Clone Dilution
CD3 AF700 Biolegend 317339 OKT3 1 in 200
CD4 BV786 Biolegend 317442 Okt4 1 in 50
CD8 BV510 Biolegend 301047 RPA-T8 1 in 400
CD45A BV650 Biolegend 304135 H100 1 in 100
CD31 PerCP-Cy5.5 Biolegend 303131 WM59 1 in 400
CD35 AF647 BD 565329 E11 1 in 50
Human Trustain FcX Fc Block Biolegend 422302 - 2.5 in 50
IL-8 FITC Biolegend 511406 E8N1 1 in 50
IL-2 PE-CY7 Biolegend 500325 MQ1-17H12 1 in 50
IL-4 PE Biolegend 500808 MP425d2 1 in 50
IL-10 BV421 Biolegend 501421 Jes3-97d 1 in 50

Panel 6: Monocyte panel
Marker Fluor Company Cat No. Clone Dilution
CD3 AF700 Biolegend 317339 OKt3 1 in 200
CD19 BV786 Biolegend 302239 HIB 19 1 in 100
CD14 BV421 Biolegend 301829 M5E2 1 in 100
CD16 PE-Cy7 Biolegend 302015 3g8 1 in 200
HLADR FITC Biolegend 307619 L243 1 in 100
CD86 PE Biolegend 305405 IT2.2 1 in 100
CD40 BV510 Biolegend 334329 5c3 1 in 100
CD1c APC Biolegend 331523 L161 1 in 100
CD56 BV650 Biolegend 362532 5.1h11 1 in 50
CD123 PerCP Cy5.5 Biolegend 306015 6H6 1 in 50
Human Trustain FcX Fc Block Biolegend 422302 - 2.5 in 50

Panel 7: Dendritic cell panel
Marker Fluor Company Cat No. Clone Dilution
CD3 AF700 Biolegend 317339 OKt3 1 in 200
CD19 BV786 Biolegend 302239 HIB 19 1 in 100
CD14 BV421 Biolegend 301829 M5E2 1 in 100
CD16 PE-Cy7 Biolegend 302015 3g8 1 in 200
CD303 FITC Biolegend 307619 L243 1 in 100
CD86 PE Biolegend 305405 IT2.2 1 in 100
CD40 BV510 Biolegend 334329 5c3 1 in 100
CD1c APC Biolegend 331523 L161 1 in 100
CD11c BV650 Biolegend 301637 3.9 1 in 50
CD123 PerCP Cy5.5 Biolegend 306015 6H6 1 in 50
Human Trustain FcX Fc Block Biolegend 422302 - 2.5 in 50

Limited functional panel for samples used in scRNAseq analysis (babies A5, A11, A12, B6 only)
Marker Fluor Company Cat No. Clone Dilution
CD4 BV786 Biolegend 317442 Okt4 1 in 50
IFNg BV650 Biolegend 502538 4S.B3 1 in 50
CD8 BV605 Biolegend 301040 RPA-T8 1 in 100
IL-2 BV510 Biolegend 500338 MQ1-17H12 1 in 50
CD56 BV421 Biolegend 362551 5.1h11 1 in 200
TCRgd PE-Cy7 Biolegend 331222 B1 1 in 50
IL-10 PE Dazzle Biolegend 501426 JES3-9D7 1 in 50
IL-4 PE Biolegend 500705 8D4-8 1 in 50
TNFa PErCP Cy5.5 Biolegend 502926 MAb11 1 in 50
IL-8 FITC Biolegend 511406 E8N1 1 in 50
CD3 AF700 Biolegend 317339 OKT3 1 in 200
IL17A AF647 Biolegend 512310 BL168 1 in 50
IL17F AF647 BD 561333 033-782 1 in 50

Human Trustain FcX Fc Block Biolegend 422302 - 2.5 in 50

Panel 8: Amphiregulin
Marker Fluor Company Cat No. Clone Dilution
CD4 BV786 Biolegend 317442 Okt4 1 in 50
CD34 BV650 Biolegend 343608 561 1 in 50
CD8 BV510 Biolegend 301047 RPA-T8 1 in 400
CD56 BV421 Biolegend 362551 5.1h11 1 in 200
TCRgd PE-Cy7 Biolegend 331222 B1 1 in 50
CD19 PE Dazzle 594 Biolegend 302252 HIB19 1 in 100
CD14 PerCP Cy5.5 Biolegend 367110 63D3 1 in 50
CD3 AF700 Biolegend 317339 OKT3 1 in 200
Amphiregulin APC eBioscience 17-5370-42 AREG559 1 in 50
IL-8 FITC Biolegend 511406 E8N1 1 in 50
IFNg PE Biolegend 502509 4S.B3 1 in 50
IL-17 BV605 Biolegend 512326 BL168 1 in 50
Human Trustain FcX Fc Block Biolegend 422302 - 2.5 in 50

Supplier / Feature barcoding antibody / Clone / Barcode sequence / Cat no / Used at:
Biolegend TotalSeq™-B0253 anti-human Hashtag 3 LNH-94, 2M2 TTCCGCCTCTCTTTG 394635 0.5 micrograms per test
Biolegend TotalSeq™-B0254 anti-human Hashtag 4 LNH-94, 2M2 AGTAAGTTCAGCGTA 394637 0.5 micrograms per test
Biolegend TotalSeq™-B0252 anti-human Hashtag 2 LNH-94, 2M2 TGATGGCCTATTGGG 394633 0.5 micrograms per test
Biolegend TotalSeq™-B0251 anti-human Hashtag 1 LNH-94, 2M2 GTCAACTCTTTAGCG 394631 0.5 micrograms per test
Biolegend TotalSeq™-B0255 anti-human Hashtag 5 LNH-94, 2M2 AAGTATCGTTTCGCA 394639 0.5 micrograms per test
Biolegend TotalSeq™-B0256 anti-human Hashtag 6 LNH-94, 2M2 GGTTGCCAGATGTCA 394641 0.5 micrograms per test
Biolegend TotalSeq™-C0251 anti-human Hashtag 1 LNH-94, 2M2 GTCAACTCTTTAGCG 394661 0.5 micrograms per test
Biolegend TotalSeq™-C0252 anti-human Hashtag 2 LNH-94, 2M2 TGATGGCCTATTGGG 394663 0.5 micrograms per test
Biolegend TotalSeq™-B0139 anti-human TCR γ/δ B1 CTTCCGATTCATTCA 331233 0.5 micrograms per test
Biolegend TotalSeq™-B0072 anti-human CD4 RPA-T4 TGTTCCCGCTCAACT 300565 0.5 micrograms per test
Biolegend TotalSeq™-B0080 anti-human CD8a RPA-T8 GCTGCGCTTTCCATT 301069 0.5 micrograms per test
Biolegend TotalSeq™-C0072 anti-human CD4 RPA-T4 TGTTCCCGCTCAACT 300567. 1 microgram per test
Biolegend TotalSeq™-C0080 anti-human CD8a RPA-T8 GCTGCGCTTTCCATT 301071. 1 microgram per test
Biolegend TotalSeq™-C0154 anti-human CD27 O323 GCACTCCTGCATGTA 302853. 1 microgram per test

Validation

All commercial antibodies were validated by their manufacturers and all represent antibodies commonly used in immunology studies. All flow cytometry antibodies were titrated using human adult PBMC prior to use within our neonatal cohorts.

Validation statements on manufacturer's websites for the companies we have purchased from:
https://www.biolegend.com/en-us/quality/quality-control
https://www.bdbiosciences.com/en-eu/products/reagents/flow-cytometry-reagents/research-reagents/quality-and-reproducibility
https://www.thermofisher.com/uk/en/home/life-science/antibodies/invitrogen-antibody-validation.html

Clinical data

Policy information about clinical studies
All manuscripts should comply with the ICMJE guidelines for publication of clinical research and a completed CONSORT checklist must be included with all submissions.

Clinical trial registration

This study was not a clinical trial

Study protocol

The study protocol was approved by the London (Chelsea) Research Ethics Committee (Ref: 15/LO/1924).

Data collection

Clinical data were collected from preterm babies on the neonatal intensive care unit at Homerton University Hospital between 2016 and 2018. In the latter part of the study, plasma samples were collected prospectively from babies on the neonatal unit at St Thomas' Hospital between June to December 2021. Seven babies, identified to have encountered one or more episodes of sepsis by neonatologists at St Thomas' were included in our study.

Outcomes

Definitions for outcome groups including 'clinical sepsis' and 'microbiologically confirmed sepsis' were modified from existing definitions published in clinical trials including the ELFIN trial (DOI:https://doi.org/10.1016/S0140-6736(18)32221-9) and nSeP trial protocols (https://bmjopen.bmj.com/content/11/12/e050100).

Plants

Seed stocks	NA
Novel plant genotypes	NA
Authentication	NA

Flow Cytometry

Plots

Confirm that:

- [x] The axis labels state the marker and fluorochrome used (e.g. CD4-FITC).
- [x] The axis scales are clearly visible. Include numbers along axes only for bottom left plot of group (a 'group' is an analysis of identical markers).
- [x] All plots are contour plots with outliers or pseudocolor plots.
- [x] A numerical value for number of cells or percentage (with statistics) is provided.

Methodology

Sample preparation	Peripheral blood mononuclear cells were extracted using Ficoll separation and cells were stored in Cryostore (Sigma) in liquid nitrogen. All samples from the same baby were batched thawed at the same time and subjected to staining with seven different flow cytometry panels as described in the methods; all antibody panels are shown in Supplementary Table 3. To assess amphiregulin by intracellular cytokine staining, a further panel was later designed and assessed in a mainly separate validation cohort of preterm babies (panel 8), though included samples from some babies in whom scRNA-seq was carried out.<br>For scRNA-seq analysis, an aliquot of PBMC were stained with Cite-seq antibodies prior to scRNA-seq. Cells were thawed, resuspended and stained as per the cell preparation protocols recommended by 10X genomics. Cell were stained with a cocktail of Totalseq-B or Total-seq C antibodies (dependent on the experiment) and hashtags to allow samples to be multiplexed together (a diagram of the scRNA-seq experimental design and list of Totalseq antibodies can be found in Supplementary Figure 15).
Instrument	BD LSRFortessa X-20
Software	BD FACSDiva Software v8.0.1 and FlowJo v10.6.2 (BD)
Cell population abundance	Cell sorting was only required for the scRNA-seq experiment in order to purify live PBMC or in one case, live CD3+ cells before library preparation. Only a fraction of the total sample was required to achieve the cell abundance required for subsequent library preparation.
Gating strategy	Gating strategies for all panels are shown in supplementary figures 9-14.

- [x] Tick this box to confirm that a figure exemplifying the gating strategy is provided in the Supplementary Information.
